# High-altitude hypoxia aggravated neurological deficits in mice induced by traumatic brain injury via BACH1 mediating astrocytic ferroptosis

**DOI:** 10.1038/s41420-025-02337-8

**Published:** 2025-02-05

**Authors:** Peng Zou, Tianjing Li, Zixuan Cao, Erwan Yang, Mingdong Bao, Haofuzi Zhang, Zhuoyuan Zhang, Dan Liu, Min Zhang, Xiangyu Gao, Junmiao Ge, Xiaofan Jiang, Zhicheng Tian, Peng Luo

**Affiliations:** 1 Department of Neurosurgery, Xijing Hospital, Fourth Military Medical University, Xi’an, 710032 China; 2 College of Life Sciences, Northwest University, Xi’an, 710069 China; 3 Department of Neurosurgery, Fuzhou 900th Hospital, Fuzhou, 350001 China

**Keywords:** Cell death in the nervous system, Brain injuries

## Abstract

Traumatic brain injury (TBI) is one of the leading causes of disability and mortality, which was classified as low-altitude TBI and high-altitude TBI. A large amount of literature shows that high-altitude TBI is associated with more severe neurological impairments and higher mortality rates compared to low-altitude TBI, due to the special environment of high-altitude hypoxia. However, the role of high-altitude hypoxia in the pathogenesis of TBI remains unclear. In order to deeply investigate this scientific issue, we constructed a high-altitude hypoxic TBI model at different altitudes and used animal behavioral assessments (Modified neurological severity score, rotarod test, elevated plus maze test) as well as histopathological analyses (brain gross specimens, brain water content, Evans blue content, hypoxia inducible factor-1α, Hematoxylin-Eosin staining and ROS detection) to reveal its underlying principles and characteristics. We found that with higher altitude, TBI-induced neurological deficits were more severe and the associated histopathological changes were more significant. Single-nuclear RNA sequencing was subsequently employed to further reveal differential gene expression profiles in high-altitude TBI. We found a significant increase in ferroptosis of astrocytes in cases of high-altitude TBI compared to those at low-altitude TBI. Analyzing transcription factors in depth, we found that Bach1 plays a crucial role in regulating key molecules that induce ferroptosis in astrocytes following high-altitude TBI. Down-regulation of Bach1 can effectively alleviate high-altitude TBI-induced neurological deficits and histopathological changes in mice. In conclusion, high-altitude hypoxia may significantly enhance the ferroptosis of astrocytes and aggravate TBI by up-regulating Bach1 expression. Our study provides a theoretical foundation for further understanding of the mechanism of high-altitude hypoxic TBI and targeted intervention therapy.

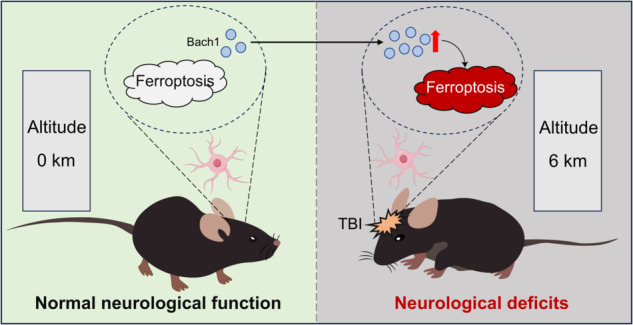

## Introduction

Traumatic brain injury (TBI) results from physical external force applied to the head, causing different degrees of damage to the skull and brain tissue. These injuries cause mechanical and persistent structural and metabolic disruptions in nerve cells, often resulting in long-term neurodegenerative conditions and accelerated aging across all age groups. This poses significant economic burden to society, as well as physical and mental burdens to patients and caregivers [1]. TBI is categorized into high-altitude and low-altitude TBI based on elevation where the injury occurs [2, 3]. High-altitude TBI refers to injuries sustained in regions above 3000 m above sea level [4]. With development of economy, tourism and societal changes, the increasing number of individuals travelling to high altitude for work or adventure tourism has made high-altitude TBI a growing concern [5, 6]. Accumulating evidence suggests that TBI at high-altitude is more severe and associated with a higher mortality rate than lower-altitude TBI, possibly due to the unique hypoxic environment at high altitudes [7]. However, the mechanism by which high-altitude hypoxia exacerbates TBI are complex and varied, and they remain incompletely understood [8]. Given the rising incidence of high-altitude TBI, therefore, understanding the pathological processes underlying hypoxia-induced aggravation of TBI is crucial [9].

Astrocytes, the most abundant and functionally complex cells in the central nervous system, play important physio-pathological roles, such as stabilizing the internal environment, releasing neurotrophic factors, and supporting neuronal function [10, 11]. Recent research has demonstrated that astrocytes after low-altitude TBI are exhibit increased reactivity, secreting numerous oxidative stress substances that disrupt energy metabolism pathways [12]. Notably, astrocytes play dual roles by secreting both harmful and reparative cytokines in response to hypoxia-induced changes [13]. The change in astrocyte numbers after high-altitude TBI remains unclear [14, 15]. Astrocytes are also implicated in mediating alterations in blood-brain barrier permeability, clearing harmful substances, and providing neuroprotection against high-altitude hypoxic TBI [16]. Nevertheless, it is unknown whether astrocytes perform more complex roles in high-altitude TBI [1].

In recent years, studies have identified that ferroptosis, a novel form of programmed cell death, as a key mechanism in the pathogenesis of neurological diseases [17, 18]. Excessive intracellular free ferrous ions (Fe^2+^) promote lipid peroxidation, and when antioxidant defenses are depleted, dysregulated polyunsaturated fatty acid oxidation in the cell membranes triggers ferroptosis [19]. Studies have demonstrated that the excess Fe^2+^ enhances reactive oxygen species (ROS) production by Fenton reactions, exacerbating cell damage [20]. Following TBI, ferroptosis generates large amounts of ROS, disrupting mitochondrial dynamics, impairing energy production, and reducing resilience to oxidative stress [21]. In addition, ferroptosis has also been involved in the destruction of energy metabolism under hypoxic conditions [22]. However, its involvement in the pathology of high-altitude hypoxia aggravating TBI remains unidentified [23].

The transcription factor Bach1 (BTB Domain And CNC Homolog 1), also known as basic leucine zipper transcription factor 1, is highly expressed in tumors such as breast and lung cancers compared to their non-tumor tissues [24, 25]. Bach1 regulates various physiological processes, including heme homeostasis, oxidative stress response, senescence, cell cycle, and mitosis [26]. In addition, studies have shown that Bach1 contributes to maintaining tumor microenvironment stability and inhibiting cancer progression by modulating ferroptosis [27]. Bach1 is also recognized as a specific regulator of hypoxia, with remarkably increased expression in mice subjected to middle cerebral artery occlusion [28]. Therefore, whether Bach1 acts as an upstream regulator in the pathogenesis of high-altitude TBI and whether it regulates ferroptosis during high-altitude hypoxia-induced TBI warrant further investigation [29].

In conclusion, this study explored the mechanisms by which high-altitude hypoxia exacerbates TBI. Using single-nuclear RNA sequencing (snRNA-seq), we identified changes in cortical protein expression profiles following high-altitude hypoxic TBI and revealed that astrocytic ferroptosis is a fundamental pathological process we identified changes in cortical. And we found Bach1 is the key molecular in the process.

## Results

### High-altitude hypoxia exacerbated neurological damage in mice induced by TBI

**Fig. 1:**
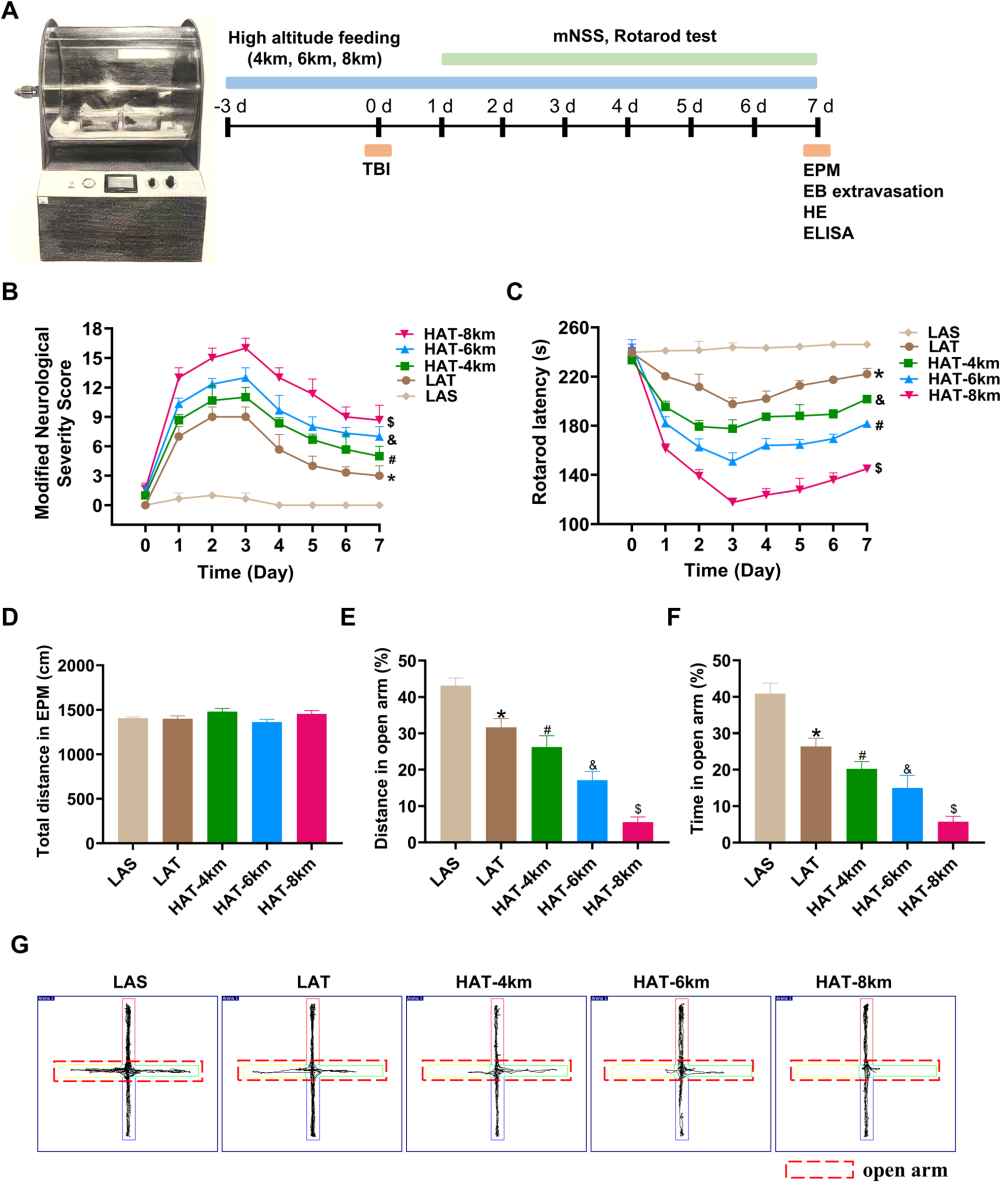
High-altitude hypoxia exacerbated neurological damage in mice induced by TBI. **A** The experimental protocol for establishment and evaluation of high-altitude hypoxic TBI model. **B** Effects of different altitudes on modified Neurological Severity Score (mNSS) in mice induced by TBI (two-way ANOVA, F = 478.9, LAT vs LAS: *P < 0.0001; HAT-4 km vs LAT: ^#^*P* < 0.0001; HAT-6 km vs HAT-4 km: ^&^*P* < 0.0001; HAT-8 km vs HAT-6 km: ^$^*P* < 0.0001) (*n* = 6). **C** Effects of different altitudes on rotarod latency in mice induced by TBI (two-way ANOVA, F = 1216, LAT vs LAS: *P < 0.0001; HAT-4 km vs LAT: ^#^*P* < 0.0001; HAT-6 km vs HAT-4 km: ^&^*P* < 0.0001; HAT-8 km vs HAT-6 km: ^$^*P* < 0.0001) (*n* = 6). **D**–**F** Effects of different altitudes on elevated plus-maze (EPM) test in mice induced by TBI. total distance in EPM test (**D**), the percentage of total distance spent in the open arms (**E**) (one-way ANOVA, F = 291.2, LAT vs LAS: *P < 0.0001; HAT-4 km vs LAT: ^#^*P* = 0.0006; HAT-6 km vs HAT-4 km: ^&^*P* < 0.0001; HAT-8 km vs HAT-6 km: ^$^*P* < 0.0001) (*n* = 6), and the percentage of total time spent in the open arms (**F**) (one-way ANOVA, F = 218.1, LAT vs LAS: *P < 0.0001; HAT-4 km vs LAT: ^#^*P* = 0.0002; HAT-6 km vs HAT-4 km: ^&^*P* = 0.0017; HAT-8 km vs HAT-6 km: ^$^*P* < 0.0001) (*n* = 6). **G** Typical trajectory plots of EPM test. Data were shown as mean ± SD.

In order to explore the potential impact of high-altitude hypoxia on the neurological damage caused by TBI, we established moderate traumatic brain injury models at varying altitudes in accordance with the experimental protocol (Fig. 1A). The modified Neurological Severity Score (mNSS) was subsequently employed to assess the neurological function of mice following different-altitude TBI. The results showed that, compared with the low-altitude sham (LAS) group, the mNSS of the low-altitude TBI (LAT) group was significantly increased, with the highest mNSS observed on the 3^rd^ day after injury (Fig. 1B). The varying altitude TBI groups, when compared with their respective sham group, also showed the same results on the 3^rd^ day after injury (Supplemental Fig. 1C–E). Above results suggests that TBI led to neurological deficits in mice at varying altitude. Further analysis of the mNSSs of LAT group, 4 km high-altitude TBI (HAT-4 km) group, 6 km high-altitude TBI (HAT-6 km) group, and 8 km high-altitude TBI (HAT-8 km) group revealed that the mNSS in the HAT-4 km group compared to LAT, HAT-6 km vs HAT-4 km and HAT-8 km vs HAT-6 km were all significantly increased (Fig. 1B), suggesting that high-altitude hypoxia exacerbated neurological deficits in mice after TBI, with the severity increasing with altitude.

To access motor function after TBI, we performed the rotarod test. The results showed that, compared with the LAS group, the rotarod latency of the LAT group was significantly decreased, with the shortest latency observed on the 3rd day after injury (Fig. 1C). Similarly, varying altitude TBI groups, compared with their respective sham group, also showed the same results on the 3^rd^ day after injury (Supplemental Fig. 1F–H). Above results from the rotarod test suggest that TBI led to motor dysfunction in mice at varying altitudes. Further analysis of the LAT, HAT-4 km, HAT-6 km and HAT-8 km groups revealed that the rotarod latency of HAT-4 km vs LAT, HAT-6 km vs HAT-4 km and HAT-8 km vs HAT-6 km were all significantly decreased (Fig. 1C), which suggested that high-altitude hypoxia aggravated motor dysfunction in mice after TBI, with motor function deteriorating incrementally as altitude increased.

In order to comprehensively assess the neurological damage of the mice after TBI, the elevated plus-maze (EPM) test was used to evaluate anxiety-like behavior. The results showed that, compared with the LAS group, the LAT group exhibited a significant decrease in both the percentage of distance and the percentage of time spent in open arm, with no difference in total distance (Fig. 1D–G). Further analysis of the LAT, HAT-4 km, HAT-6 km, and HAT-8 km groups revealed that the percentage of distance and time spent in open arm of the HAT-4 km vs LAT, HAT-6 km vs HAT-4 km and HAT-8 km vs HAT-6 km were all significantly decreased, with no difference in total distance (Fig. 1D–G). These findings suggest that high-altitude hypoxia exacerbates anxiety-like behavior in mice after TBI, with the severity of anxiety increasing incrementally as altitude rises. The above results indicate that, under the same injury intensity, higher altitudes correlate with poorer motor function and more pronounced anxiety-like behavior in TBI mice. This suggests that high-altitude hypoxia exacerbates neurological damage in mice induced by TBI.

### High-altitude hypoxia exacerbated histopathological injury in mice induced by TBI

**Fig. 2:**
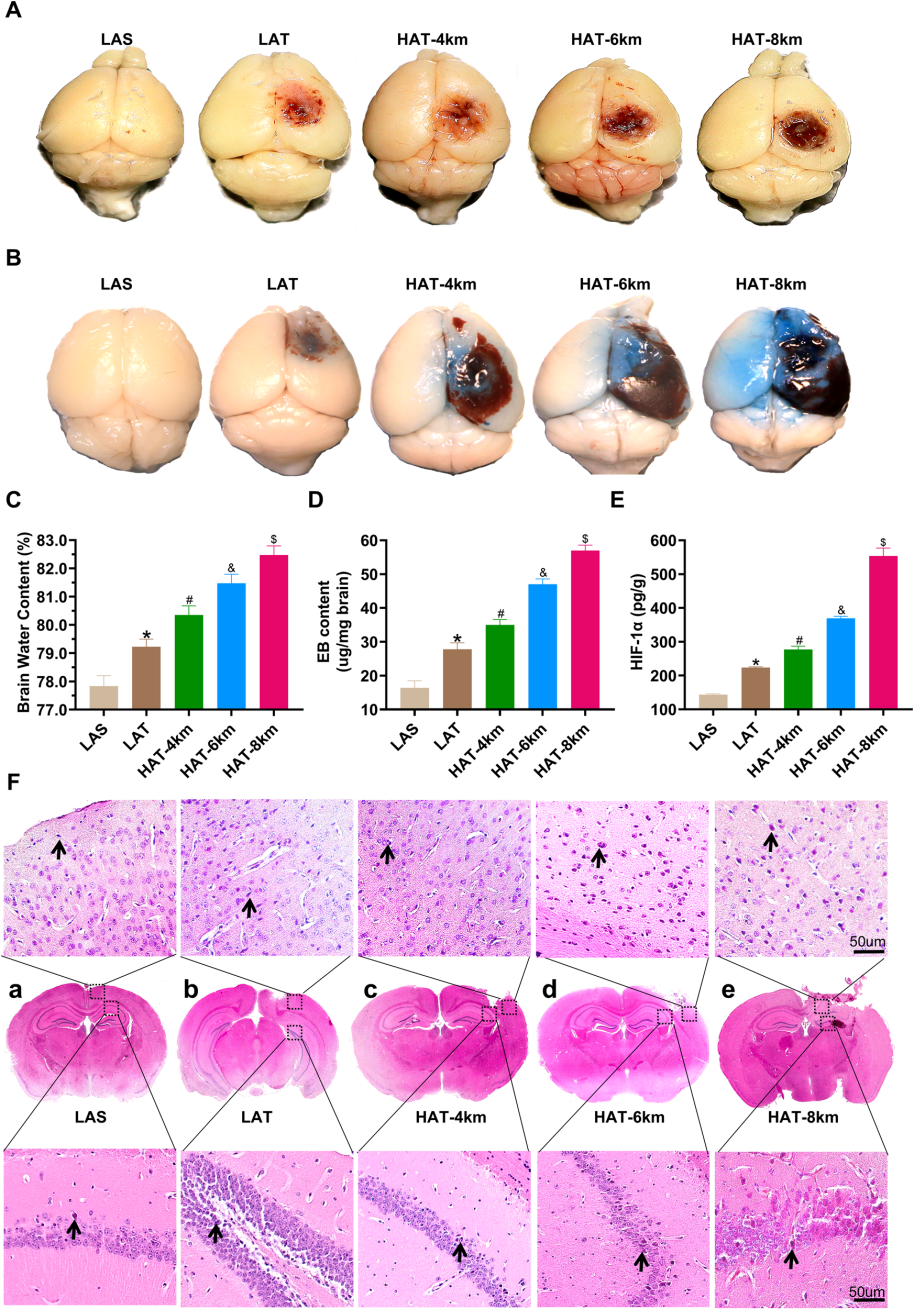
High-altitude hypoxia exacerbated histopathological injury in mice induced by TBI. **A** The brain gross specimens following different-altitude TBI. **B** Evans blue (EB) extravasation test. The brain gross specimens through tail vein injection of Evans blue. **C** The brain water content of different-altitude TBI (one-way ANOVA, F = 257.5, LAT vs LAS: *P < 0.0001; HAT-4 km vs LAT: ^#^*P* < 0.0001; HAT-6 km vs HAT-4 km: ^&^*P* < 0.0001; HAT-8 km vs HAT-6 km: ^$^*P* < 0.0001) (*n* = 6). **D** The EB content of different-altitude TBI (one-way ANOVA, F = 408.2, LAT vs LAS: *P < 0.0001; HAT-4 km vs LAT: ^#^*P* < 0.0001; HAT-6 km vs HAT-4 km: ^&^*P* < 0.0001; HAT-8 km vs HAT-6 km: ^$^*P* < 0.0001) (*n* = 6). **E** The content of HIF-1α of different-altitude TBI (one-way ANOVA, F = 541.6, LAT vs LAS: *P < 0.0001; HAT-4 km vs LAT: ^#^*P* < 0.0001; HAT-6 km vs HAT-4 km: ^&^*P* < 0.0001; HAT-8 km vs HAT-6 km: ^$^*P* < 0.0001) (*n* = 6). **F** HE staining of the right side of hippocampus and cortex in different groups, scale = 50 μm. Data were shown as mean ± SD.

To investigate the possible effect of high-altitude hypoxia on histopathological injury induced by TBI, we performed several assessments, including brain gross specimen collection, Evans blue (EB) extravasation testing, HIF-1α content examination, and hematoxylin-eosin (HE) staining. The brain gross specimens from different-altitude TBI groups showed varying degrees of contusion, bleeding, and edema in the damaged area, with blood extending into the subarachnoid space (Fig. 2A). Compared to the LAS group, the LAT group exhibited a significant increase in hemorrhage volume. Moreover, the HAT-4 km group vs LAT, HAT-6 km vs HAT-4 km and HAT-8 km vs HAT-6 km groups all presented a comparable increasing trend in hemorrhage volume (Fig. 2A). Brain gross specimens from the HAS-4 km, HAS-6 km and HAS-8 km groups are shown in Supplemental Fig. 2A. In order to assess brain edema, we calculated brain water content. Further quantitative results demonstrated that, compared with the LAS group, the LAT group exhibited significantly increased brain water content (Fig. 2C). Varying altitude TBI groups showed the same trend compared to their respective sham groups (Supplemental Fig. 2D–F). Similarly, the brain water content of HAT-4 km vs LAT, HAT-6 km vs HAT-4 km and HAT-8 km vs HAT-6 km groups were all dramatically elevated, suggesting that as the altitude increased, brain tissue water content gradually increased after TBI (Fig. 2C). To assess BBB integrity, we performed the EB extravasation test (Fig. 2B and Supplemental Fig. 2B). As expected, the EB content in the brain tissue was significantly higher in the TBI groups compared to their respective sham groups (Supplemental Fig. 2G–I). The results showed that, with increasing altitude, more Evans blue infiltrated brain tissue, indicating more severe BBB damage (Fig. 2B, D). These findings suggests that high-altitude hypoxia exacerbates BBB damage caused by traumatic brain injury.

To assess tissue hypoxia, we further measured protein levels of HIF-1α, a well-established marker of hypoxia [30]. The results demonstrated that, compared with the LAS group, the LAT group showed significantly elevated HIF-1α levels, indicating that TBI induces brain tissue hypoxia (Fig. 2E). Meanwhile, the HIF-1α protein levels were significantly higher in the HAT-4 km vs LAT, HAT-6 km vs HAT-4 km and HAT-8 km vs HAT-6 km groups, indicating that higher altitudes lead to more severe hypoxic in brain tissue (Fig. 2E). HE staining was used to observe the morphology of cortical and hippocampal neurons. No damage was observed in the LAS group under low magnification, and the neurons appeared neatly arranged under high magnification (Fig. 2Fa). Similar results were found in the HAS-4km, HAS-6km and HAS-8km groups (Supplemental Figure 2Ca–c). Compared with the LAS group, the LAT group showed areas of damage with a higher number of necrotic neurons. Hippocampal neurons presented slight swelling and took on a circular or elliptical shape (Fig. 2Fb). In the HAT-4km group, the damage area was larger, with more pronounced neuronal edema and a small number of hippocampal cells featuring deeply stained and shrunken nucleus (Fig. 2Fc). The HAT-6km group showed a significant increase in neuronal fragments and a large number of hippocampal neurons with abnormal morphology, changing from a normal round or oval shape to a spindle shape (Fig. 2Fd). The HAT-8km group displayed even more severe hemorrhage, with more cellular debris and erythrocytes visible in hippocampal neurons (Fig. 2Fe). These experimental results suggest that TBI induces BBB disruption, cerebral edema, tissue hypoxia and neuronal damage in the cortex and hippocampus. Moreover, high-altitude hypoxia exacerbates these pathological changes, with damage becoming more pronounced as altitude increases.

We evaluated the survival rate of TBI mice at different altitudes and found that higher altitudes were associated with lower survival rates (Supplemental Fig. 1I). Based on these findings, a 6 km high-altitude condition was adopted for subsequent experiments to facilitate further study.

### Single-nuclear RNA sequencing (snRNA-seq) analysis revealed alterations of neuron in cortex after high-altitude TBI

**Fig. 3:**
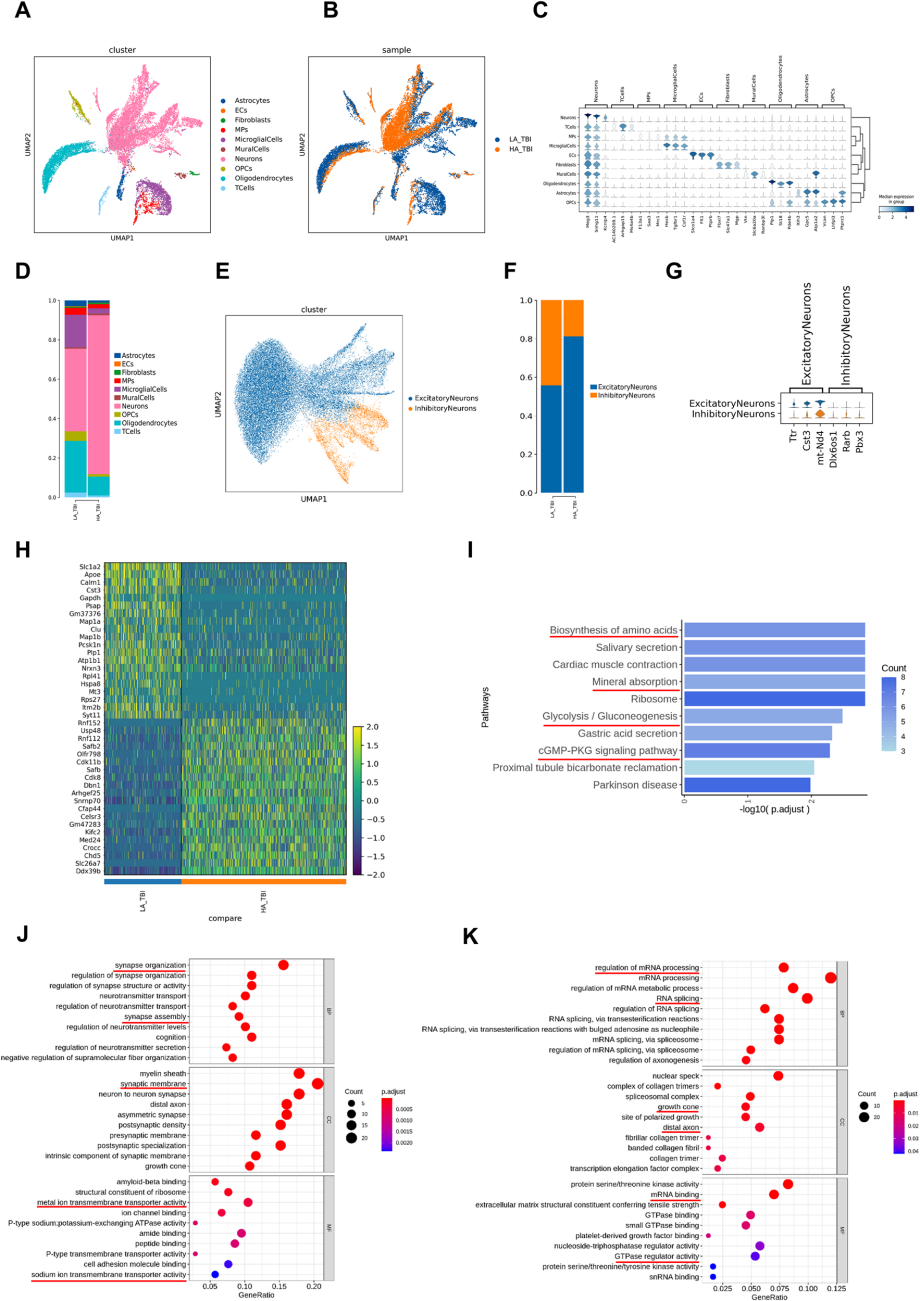
SnRNA-seq analysis revealed alterations of neuron in cortex after high-altitude TBI. **A**, **B** UMAP plot of 17,002 high-quality cells to show 10 main cell-types based on the expression of known marker genes, colored by cell type and cell origin respectively. **C** Expression of representative marker genes for each cell type. Gene expression violin plots are shown in log-scale Unique Molecular Identifiers (UMI). **D** The ratio of each cell type in LA-TBI and HA-TBI groups. **E** UMAP plot of neurons to show excitatory-neurons and inhibitory-neurons. **F** The ratio of two types neurons in LA-TBI and HA-TBI groups. **G** Expression of representative marker genes for excitatory-neurons and inhibitory-neurons. **H** The heatmap of DEGs in excitatory-neurons between two groups. **I** The KEGG pathways of up-regulated DEGs in excitatory-neurons. **J**, **K** The top 10 GO enrichments in BP, CC, MF in up-regulated (**J**) and down-regulated (**K**) DEGs. Each node signaled a GO term, and its size represented the gene number.

To reveal the cellular and molecular dynamics of TBI under high-altitude hypoxia, we collected brain tissues from the injured areas of the 2 groups of mice (LA_TBI and HA_TBI) and performed snRNA-seq analysis. The snRNA-seq data from 17,002 quality-controlled cells were analyzed using unsupervised clustering via uniform manifold approximation and projection (UMAP) to resolve cell-type composition. Based on known cell-type-specific markers and transcriptional features, we identified 10 distinct cell types in both the LA_TBI and HA_TBI groups: astrocytes, endothelial cells (ECs), fibroblasts, mononuclear phagocytes (MPs), microglial cells, mural cells, neurons, oligodendrocytes precursor cells (OPCs), oligodendrocytes, and T cells (Fig. 3A, B). There were no differences in the subpopulations between the two groups. The top three genes expressed in each subpopulation, considered specific marker genes, were used for identification and comparison with other subpopulations (Fig. 3C). We found that the proportion of neurons was significantly higher in the HA_TBI group compared to the LA_TBI group, while the proportion of other cells decreased (Fig. 3D, Table 1), suggesting an increase death of non-neuronal cells in high-altitude TBI.

**Table 1:** Different proportions between the LA_TBI and HA_TBI group.

cluster	LA_TBI	HA_TBI
Astrocytes	276	82
ECs	25	6
Fibroblasts	55	56
MPs	343	158
MicroglialCells	1578	196
MuralCells	68	64
Neurons	4012	6007
OPCs	462	96
Oligodendrocytes	2508	708
TCells	230	72

We further subdivided the neuronal cells into two types based on known cell type-specific markers (Fig. 3E, G). The proportion of excitatory neurons was elevated in the HA_TBI group (Fig. 3F). The top 20 up-regulated differentially expressed genes (DEGs) and top 20 down-regulated DEGs in excitatory neurons between the two groups are shown in Fig. 3H. GO analysis revealed that the upregulated DEGs in excitatory neurons were enriched in some pathways associated with synapse and transmembrane transporter (Fig. 3J). Conversely, the downregulated DEGs were enriched in mRNA processing, GTPase regulator activity, and axon associated processes (Fig. 3K). The KEGG pathway analysis indicated significant enrichment of pathways related to mineral absorption, glycolysis/gluconeogenesis and cGMP-PKG signaling pathway in the excitatory neurons of the high-altitude TBI group (Fig. 3I). Additionally, we showed the DEGs of inhibitory neurons between the two groups (Supplemental Fig. 3A). GO analysis revealed that the upregulated DEGs of the inhibitory neurons enriched in axon and synapse associated processes (Supplemental Fig. 3B). The downregulated DEGs were enriched in the regulation of the neuron apoptotic process, GTPase regulator activity, and other functions (Supplemental Fig. 3C). However, there was no significant difference in the KEGG pathways between the two groups of inhibitory neurons. Taken together, these results suggest that the changes of neurons in the HA_TBI are mainly associated with neuronal structure and function.

### SnRNA-seq analysis revealed that Bach1 might be a key molecule participating ferroptosis in astrocytes after high-altitude TBI

**Fig. 4:**
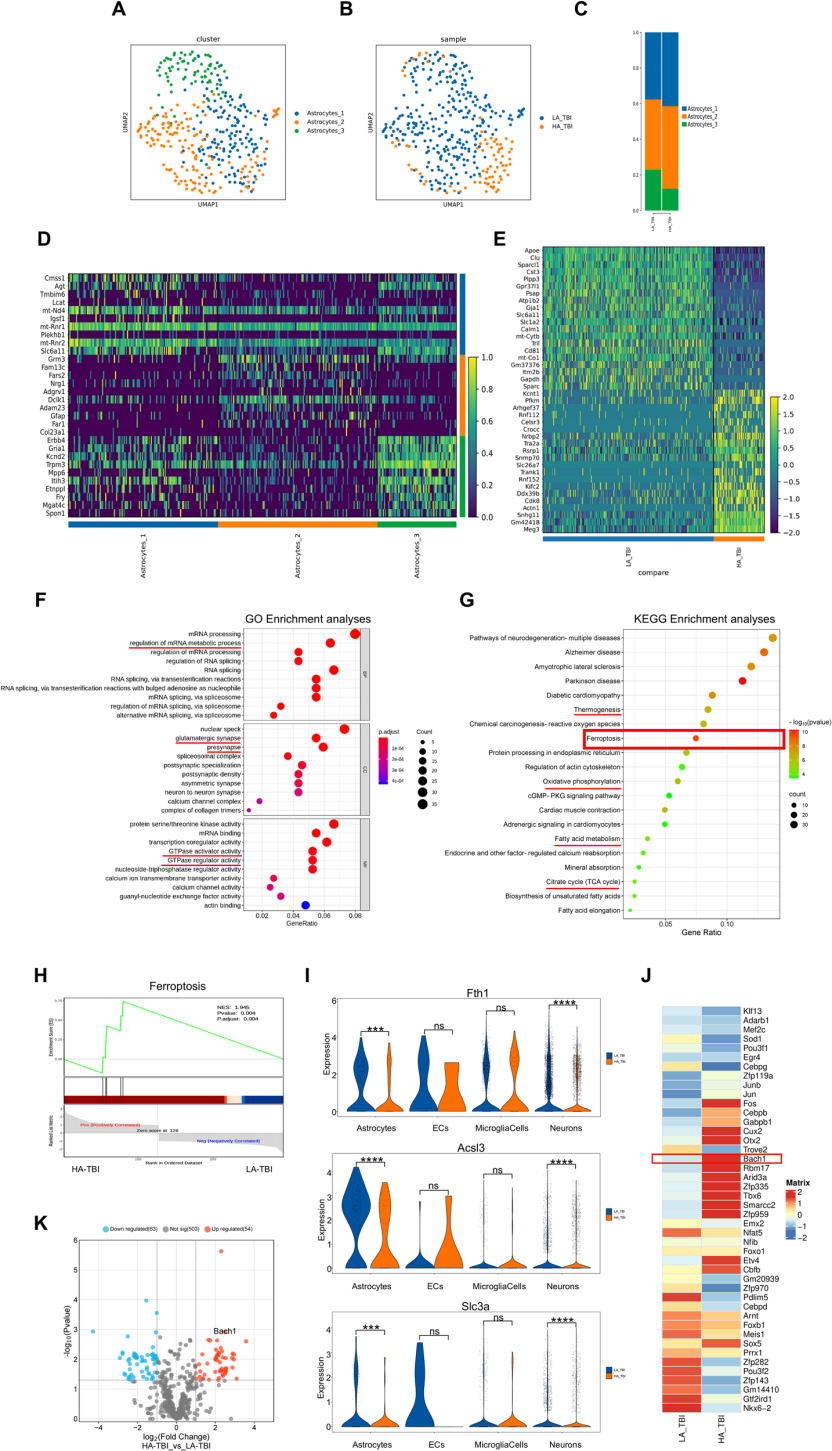
SnRNA-seq analysis revealed that Bach1 might be a key molecule participating ferroptosis in astrocytes after high-altitude TBI. **A** UMAP showing the clustering of astrocytes subsets based on the expression of marker genes. **B** UMAP showing the astrocytes from LA-TBI and HA-TBI groups. Each dot corresponds to one single cell colored according to cell cluster. **C** Demonstration of the ratio of the 3 astrocytes subpopulations in LA-TBI and HA-TBI groups. **D** Representative molecular signatures for astrocytes subsets. **E** Heatmap plots showing representative differentially expressed genes between the LA-TBI and HA-TBI groups. Per group *n* = 3. **F** The top 10 GO enrichments in BP, CC, MF. Each node signaled a GO term, and its size represented the gene number. The color indicates the *P*-value. **G** KEGG enrichment analysis on astrocytic population, showing upregulated pathways. **H** GSEA showing ferroptosis pathway enriched in astrocytes induced by HA-TBI and LA-TBI. NES, normalized enrichment score. **I** Violin plots for selected genes: Fth1, Acsl3, Slc3a. **J** SCENIC analyses in the LA-TBI and HA-TBI groups (***P < 0.001, ****P < 0.0001 between LA-TBI and HA-TBI. **K** Volcano plot of upregulated and downregulated genes in the LA-TBI and HA-TBI groups. Data were shown as mean ± SD.

Based on the essential role of astrocytes in regulating tissue hypoxia and damage repair, we next focused on astrocytic populations. A total of 358 astrocytes were identified from both groups. Further clustering of astrocytes revealed three distinct subtypes (Fig. 4A), annotated as: Astrocytes_1, Astrocytes_2 and Astrocytes_3, according to their gene expression profiles (Fig. 4D). Both the LA_TBI and HA_TBI groups had the same subtypes (Fig. 4B) with similar composition (Fig. 4C). However, consistent with the decreased proportion of astrocytes in the HA_TBI group, we considered that high-altitude TBI results in a reduction in the quantity of all three astrocyte subsets, aligning with the snRNA-seq findings (Table 2). To further explore these changes, we analyzed the top 20 up-regulated DEGs and 20 down-regulated DEGs in astrocytes between LA_TBI and HA_TBI groups (Fig. 4E). Above results of snRNA-seq analysis indicate that the number of astrocytes significantly decreased in high-altitude TBI compared to low-altitude TBI, accompanied by altered gene expression profiles.

**Table 2:** High-altitude TBI results in a reduction in the quantity of all three astrocyte subsets.

cluster	LA_TBI	HA_TBI
Astrocytes_1	104	34
Astrocytes_2	109	38
Astrocytes_3	63	10

To further analyze the characteristics of the differentially expressed genes in astrocytes after high-altitude TBI, we conducted GO and KEGG analyses. GO analysis revealed that up-regulated DEGs in astrocytes from the HA_TBI group enriched in some processes associated with mRNA, synapse and GTPase (Fig. 4F). The KEGG pathway enrichment analysis indicated up-regulated DEGs significant enriched in pathways related to neurodegenerative disease (Alzheimer disease and Parkinson disease), thermogenesis, ferroptosis, oxidative phosphorylation, fatty acid metabolism, and the citrate cycle (Fig. 4G). The KEGG analysis showed up-regulated DEGs in astrocytes were mainly related to energy metabolism, which showed that altered energy metabolism in astrocytes plays a crucial role in the exacerbation of TBI under high-altitude hypoxic conditions. Ferroptosis, a form of regulated cell death closely associated with energy metabolism, oxidative phosphorylation, and neurodegenerative diseases, was significantly enriched in the KEGG enrichment analysis of astrocytes (Fig. 4G). This suggests that ferroptosis may mediate the alterations in astrocyte energy metabolism in TBI under high-altitude hypoxia.

To investigate the role of ferroptosis in astrocytes further, we performed the Gene Set Enrichment Analysis (GSEA), which showed a significant increase in the enrichment of the ferroptosis pathway in astrocytes from the HA-TBI group compared with LA-TBI group (Fig. 4H). Further statistical analysis of ferroptosis markers, such as Fth1 (which defends against oxidation and protects cells from free radical damage), Acsl3 (which inhibits ferroptosis), and Slc3a (with antioxidant effects), showed significant increases in their expression in astrocytes from the HA_TBI group (Fig. 4I). We also analyzed the these ferroptosis markers in ECs, microglial cells and neurons. These results showed that these markers were significantly increased in neurons, but expression was extremely low in ECs and microglial cells (Fig. 4I). The above analysis results suggest that the ferroptosis affects astrocytes and neurons under high-altitude hypoxic TBI. In order to deeply analyze the key molecules affecting ferroptosis in high-altitude hypoxic TBI, we performed the transcription factor analysis between the HA-TBI and LA-TBI groups using SCENIC (single-cell regulatory network inference and clustering). This analysis revealed that 179 transcription factors were up-regulated and 191 were down-regulated. Notably, the transcription factor Bach1, which has been highly associated with ferroptosis in acute lung injury and lumbar disc herniation, was significantly elevated in the HA-TBI group (Fig. 4J) and prominently featured in the upregulated region of the differential gene volcano plot for astrocytes (Fig. 4K). These results suggest that Bach1 may be a key molecule mediating ferroptosis in high-altitude hypoxic TBI. Taken together, we found that the energy metabolism of astrocytes was significantly altered in TBI mice under conditions of high-altitude hypoxia compared to low-latitude, potentially contributing to the exacerbation of TBI. Further studies revealed that the ferroptosis pathway was involved in these changes, with the transcription factor Bach1 emerging as a key molecule. The specific role of Bach1 in this process warrants further verification.

### Alteration of Bach1 expression and ferroptosis in astrocytes after high-altitude TBI

**Fig. 5:**
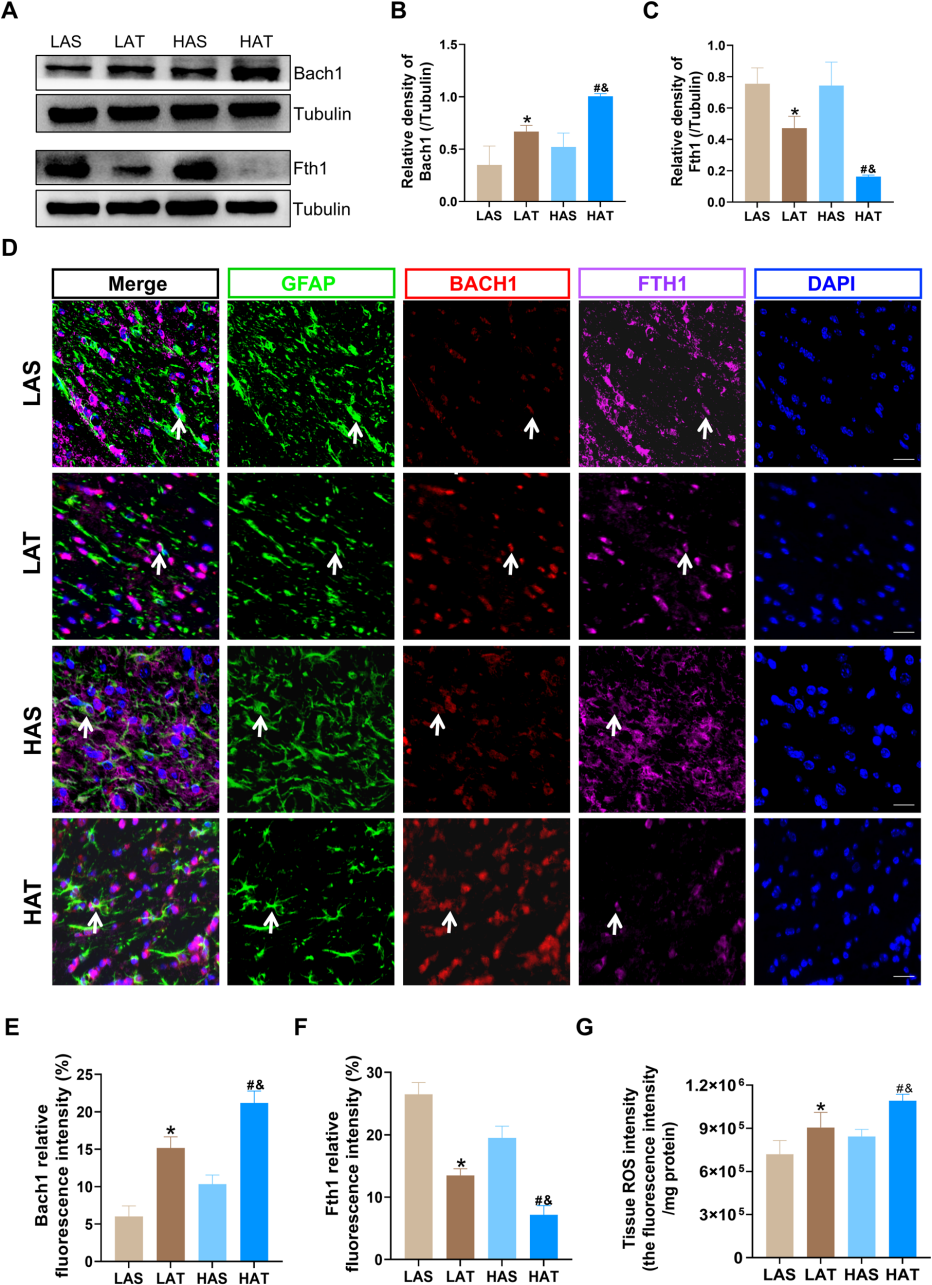
Alteration of Bach1 expression and ferroptosis in astrocytes after high-altitude TBI. **A**–**C** Western blotting detection of the protein level of Bach1 (one-way ANOVA, F = 17.16, LAT vs LAS: *P = 0.0407; HAT vs HAS: ^#^*P* = 0.0041; HAT vs LAT: ^&^*P* = 0.0309), Fth1 (one-way ANOVA, F = 24.24, LAT vs LAS: *P = 0.0310; HAT vs HAS: ^#^*P* = 0.0004; HAT vs LAT: ^&^*P* = 0.0207) in the LAS, LAT, HAS and HAT groups (per group *n* = 3). **D**–**F** Representative images in astrocytes of different groups demonstrated Bach1 (one-way ANOVA, F = 124.3, LAT vs LAS: *P < 0.0001; HAT vs HAS: ^#^*P* < 0.0001; HAT vs LAT: ^&^*P* < 0.0001) was significantly increased and the ferroptosis marker Fth1 (one-way ANOVA, F = 159.7, LAT vs LAS: *P < 0.0001; HAT vs HAS: ^#^*P* < 0.0001; HAT vs LAT: ^&^*P* < 0.0001) was significantly decreased in high-altitude TBI (per group *n* = 3). **G** Tissue ROS intensity (one-way ANOVA, F = 15.62, LAT vs LAS: *P < 0.0260; HAT vs HAS: ^#^*P* < 0.0036; HAT vs LAT: ^&^*P* < 0.0253) in the LAS, LAT, HAS and HAT groups (per group *n* = 4). Scale bar: 20 μm. Data were shown as mean ± SD.

The snRNA-seq results showed that transcription factor Bach1 was highly expressed in the astrocytes from the HA_TBI group, while Fth1, a marker of ferroptosis, was significantly reduced. To verify these findings, we conducted western blot and immunofluorescence staining experiment in vivo. Western blot analysis showed that the protein level of Bach1 was distinctly elevated in both the LA_TBI and HA_TBI groups compared to their respective sham groups, with a marked increase in Bach1 expression in the HA_TBI group compared to LA_TBI group (Fig. 5A, B). On the contrary, the protein level of Fth1 was decreased in both the LA_TBI and HA_TBI groups compared to their respective sham group, with a further significant reduction in the HA_TBI group compared with LA_TBI group (Fig. 5A, C). These findings predict that TBI triggers an increase in Bach1 expression and a decrease in Fth1 expression, and that high-altitude hypoxia exacerbates this effect by further elevating Bach1 expression and further diminishing Fth1 expression, suggesting that high-altitude hypoxia can aggravate the pathological changes caused by TBI. Immunofluorescence staining of GFAP, Bach1 and Fth1 further revealed the relationship between astrocytes and ferroptosis after TBI under conditions of high-altitude hypoxia. Consistent with the WB results, the increased fluorescence intensity of Bach1 and decreased fluorescence intensity of Fth1 were observed in both the LA_TBI and HA_TBI groups compared to their respective sham groups (Fig. 5D–F). Furthermore, the HA_TBI group exhibited significantly higher fluorescence intensity of Bach1, the significantly lower fluorescence intensity for Fth1 compared to LA_TBI group (Fig. 5D–F). To confirm that ferroptosis resulted in detrimental outcomes, we evaluated ROS levels using a tissue ROS detection kit. The results showed a marked increase in the production of ROS in both the LA_TBI and HA_TBI groups compared with their respective sham groups, with significantly higher ROS levels in the HA_TBI group compared with LAT group (Fig. 5G). This suggests that high-altitude hypoxia induces ferroptosis, leading to increased ROS production. Collectively, our data demonstrate that Bach1 expression was significantly increased and the ferroptosis marker Fth1 was significantly decreased in high-altitude TBI model, suggesting that Bach1 may play a role in the pathogenesis of high-altitude TBI through ferroptosis in astrocytes.

### Knockdown of Bach1 could inhibit ferroptosis of astrocytes and improve neurological deficits induced by high-altitude TBI in mice

**Fig. 6:**
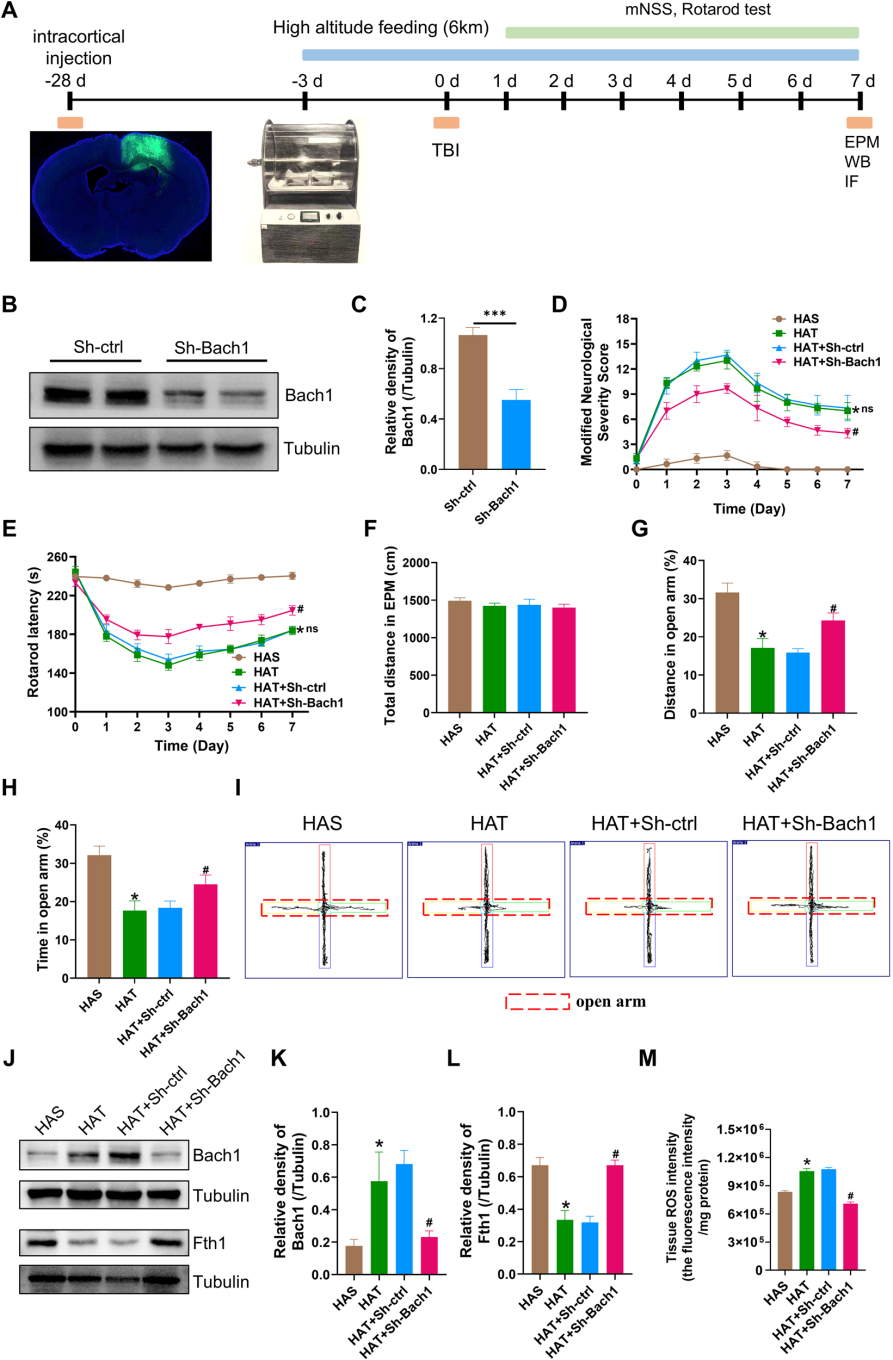
Knockdown of Bach1 could inhibit ferroptosis of astrocytes and improve neurological deficits induced by high-altitude TBI in mice. **A** Experimental design. **B**, **C** Western blot was used to verify knockdown efficiency (*n* = 3) (unpaired t-test, t = 8.725, *P* = 0.0010). **D** Bach1 konckdown could alleviate the neurological deficit caused by high-altitude TBI (*n* = 6) (two-way ANOVA, F = 539.4, HAT vs HAS: *P < 0.0001; HAT-sh-ctrl vs the HAT: ^ns^*P* = 0.6116; HAT-sh-Bach1 vs HAT-sh-ctrl: ^#^*P* < 0.0001). **E** Bach1 knockdown could alleviate motor dysfunction caused by high-altitude TBI (*n* = 6) (two-way ANOVA, F = 782.6, HAT vs HAS: *P < 0.0001; HAT-sh-ctrl vs the HAT: ^ns^*P* = 0.3790; HAT-sh-Bach1 vs HAT-sh-ctrl: ^#^*P* < 0.0001). **F**–**H** Bach1 knockdown could alleviate anxiety-like behavior caused by high-altitude TBI. total distance in EPM test (**F**), the percentage of total distance spent in the open arms (**G**) (one-way ANOVA, F = 101.2, HAT vs HAS: *P < 0.0001; HAT vs HAT-sh-ctrl: *P* = 0.6182; HAT-sh-ctrl vs HAT-sh-Bach1: ^#^*P* < 0.0001), and the percentage of total time spent in the open arms (H) (one-way ANOVA, F = 68.17, HAT vs HAS: *P < 0.0001; HAT vs HAT-sh-ctrl: *P* = 0.9143; HAT-sh-ctrl vs HAT-sh-Bach1: ^#^*P* < 0.0001). **I** Typical trajectory plots of EPM test. **J**–**L** Western blotting detection of the protein level of Bach1 (one-way ANOVA, F = 17.62, HAT vs HAS: *P = 0.0064; HAT vs HAT-sh-ctrl: *P* = 0.6134; HAT-sh-ctrl vs HAT-sh-Bach1: ^#^*P* = 0.0031), Fth1 (one-way ANOVA, F = 58.84, HAT vs HAS: *P < 0.0001; HAT vs HAT-sh-ctrl^:^ *P* = 0.9719; HAT-sh-ctrl vs HAT-sh-Bach1: ^#^*P* < 0.0001) in the HAS, HAT, HAT+sh-ctrl and HAT+sh-Bach1 groups (per group *n* = 3). **M** Tissue ROS intensity (one-way ANOVA, F = 324.5, HAT vs HAS: *P < 0.0001; HAT vs HAT-sh-ctrl: *P* = 0.4469; HAT-sh-ctrl vs HAT-sh-Bach1: ^#^*P* < 0.0001) in the HAS, HAT, HAT+sh-ctrl and HAT+sh-Bach1 groups (per group *n* = 4). Data were shown as mean ± SD.

To verify whether Bach1 was involved in the pathological process of high-altitude hypoxic TBI by mediating ferroptosis, we regulated the expression of Bach1 to detect animal behavior and changes in astrocytic ferroptosis in accordance with the experimental protocol (Fig. 6A). The expression of Bach1 was intervened by injecting adeno-associated viruses (rAAV2/5-GFAP-EGFP-5′miR-30a-shRNA Bach1-3′miR-30a-WPREs) into right cerebral cortex. The fluorograms depicting viral expression showed that the virus was successfully transfected (Fig. 6A). After 28 days, western blot was used to verify knockdown efficiency (Fig. 6B). As can be seen, Bach1 was obviously declined in the sh-Bach1 group compared with sh-ctrl group (Fig. 6B, C). Behavioural tests were performed on HAS, HAT, HAT+sh-ctrl, and HAT+sh-Bach1 mice after TBI modelling. The mNSS score results showed that the score of the HAT group was significantly higher than that of the HAS group (Fig. 6D). Whereas in the absence of significant differences between the HAT group and the HAT+sh-ctrl group, the score of the HAT+sh-Bach1 group was significantly lower than that of the HAT+sh-ctrl group, indicating that the intervention of Bach1 could alleviate the neurological deficit caused by high-altitude hypoxic TBI (Fig. 6D). The rotarod latency of the HAT group substantially declined than that of the HAS group (Fig. 6E). Under the absence of significant differences between the HAT group and the HAT+sh-ctrl group, the rotarod latency of the HAT+sh-Bach1 group significantly rised than that of the HAT+sh-ctrl group, illustrating that Bach1 knockdown could alleviate motor dysfunction caused by high-altitude hypoxic TBI (Fig. 6E). The EPM test results demonstrated compared with the HAS group, the percentage of distance and the percentage of time in open arm in the HAT group were both significantly decreased (Fig. 6G–I). In the case of no significant difference between the HAT group and the HAT+sh-ctrl group, the percentage of distance and the percentage of time in open arm in the HAT+sh-Bach1 group significantly rised than that of the HAT+sh-ctrl group (Fig. 6G–I). Besides, all of these groups had no difference in total distance (Fig. 6F). The EPM test results implied Bach1 knockdown could alleviate anxiety-like behavior caused by high-altitude hypoxic TBI.

Simultaneously, to verify the improvement of histopathological injury after Bach1 knockdown in high-altitude hypoxic TBI, western blot were performed. The western blot results documented that the obviously elevated Bach1 expression, the obviously declined Fth1 expression were in the HAT group compared to that in the HAS group (Fig. 6J–L). In the absence of significant differences between the HAT group and the HAT+sh-ctrl group, HAT+sh-Bach1 group significantly mitigated Bach1 expression and elevated Fth1 expression than that of the HAT+sh-ctrl group, illustrating that depletion of Bach1 significantly reversed the increases in astrocytic ferroptosis induced by high-altitude hypoxic TBI. In addition, the obviously elevated ROS were in the HAT group compared to that in the HAS group (Fig. 6M). In the absence of significant differences between the HAT group and the HAT+sh-ctrl group, HAT+sh-Bach1 group significantly mitigated ROS than that of the HAT+sh-ctrl group, illustrating that depletion of Bach1 significantly reversed the increases of ROS induced by high-altitude hypoxic TBI. The above results indicated that Bach1 knockdown could effectively reversed astrocytic ferroptosis and alleviate the neurological deficits induced by high-altitude hypoxic TBI, suggesting that Bach1 may be a key molecule mediating the astrocytic ferroptosis induced by high-altitude hypoxic TBI, contributing to the aggravation of neurological deficit.

## Discussion

High-altitude hypoxic TBI represents a unique region in the field of medicine and has attracted increasing attention [31]. The aim of our study was to identify potential targets for high-altitude hypoxic TBI intervention and treatment by investigating the underlying molecular mechanism of high-altitude hypoxia aggravating TBI through snRNA-seq analyses. First, we found that high-altitude hypoxia exacerbated neurological functions and histological lesions at 1 week after TBI, with the higher the altitude, the more significant the damage. Then, we showed that energy metabolic pathways in astrocyte was altered after high-altitude hypoxic TBI, and that these energy metabolic pathways were probably associated with neurodegenerative diseases, thermogenesis, and ferroptosis, suggesting that astrocytic ferroptosis persisted in the injured brain tissue after high-altitude hypoxic TBI. Transcription factor analysis also identified Bach1 as a core molecule participating in high-altitude hypoxic TBI pathology. Bach1 knockdown could effectively ameliorated neurological deficits induced by high-altitude hypoxic TBI. Overall, our findings suggest that Bach1 could be an important target for treating neurological deficits after high-altitude hypoxic TBI.

SnRNA-seq is an important tool and method for investigating the molecular pathology of neurological diseases, and has been applied to various diseases, such as stroke and neurodegeneration [32]. Numerous studies have used a SnRNA-seq approach to analyze the mechanisms of TBI in plain area and have identified a variety of potential biological markers and intervention targets [33]. However, the pathogenesis of high-altitude hypoxic TBI may involve different alterations at the molecular level compared with TBI in plain area [34, 35]. Currently, previous research on high-altitude hypoxic TBI is mainly limited to some mechanisms which have been adequately demonstrated in plains TBI, such as hyperbaric oxygen preconditioning, application of neuroprotective agents and inhibition of inflammatory factors [36]. A previous study identified that high altitude aggravated TBI significantly and hyperbaric oxygen pre-conditioning could attenuate TBI in rats at high altitude by improvement of brain tissue oxygen pressure [37]. Another study substantiated that high altitude aggravated brain damage after TBI, and that the damage also increased with increasing altitude, and that L-serine, an endogenous amino acid, might be a neuroprotective agent against high-altitude hypoxic TBI [9, 38]. Even though these results are partially consistent with our findings, the potential molecular mechanisms of high-altitude hypoxia aggravating TBI are still missing [39, 40]. Therefore, we performed a SnRNA-seq analysis of the injured brain tissue from high-altitude hypoxic TBI. To our knowledge, this is the first time that brain tissue from high-altitude hypoxic TBI has been analysed using snRNA-seq technology. Previous snRNA-seq analyse of brain tissue from TBI in plain area confirmed that an expanded astrocyte population which was significantly activated was responsible for inflammation in the acute phase of TBI [41, 42]. The other snRNA-seq analyse of TBI in plain area verified that the restoration of metabolic pathways within astrocytes reversed cognitive impairment caused by TBI [43]. These results were consistent with our findings, which all indicated that astrocytes had a critical role in trauma and hypoxia repair [42]. Similar to previous studies, we found that the oxidative phosphorylation signaling pathway, fatty acid metabolism, mineral absorption, citrate cycle (TCA cycle), and biosynthesis of unsaturated fatty acids were important in high-altitude hypoxic TBI. We also identified new biological processes from GO enrichment, such as proteasome-mediated ubiquitin-dependent protein catabolic process and vesicle-mediated transport in synapse, which were significantly linked to neurodegenerative diseases and energy metabolism induced by ferroptosis.

In recent years, omics have gained prominence in the investigation of TBI, including proteomics and phosphoproteomics. The latest two investigations conducted by our research team employed proteomics to analyze the brain tissue of mice following plain TBI. One of these studies demonstrated that the hippocampal proteomic analysis revealed phosphorylation of NR1 was crucial in mediating cognitive impairment induced by rmTBI [44]. The other study revealed a map of early and late-stage protein networks in TBI, and β-catenin independent Wnt signaling and small GTPase mediated signaling transmission pathway were the most important interacting proteins in the early stage of TBI [45], which was consistent with snRNA-seq of high-altitude TBI in our study. So far, the proteomics of plateau TBI has not been reported. The primary advantage of utilizing single-cell transcriptomics technology in our investigation revolved a comprehensive response to which genes were activated or repressed, which was critical for understanding cell function, disease mechanisms, etc., However, although proteomics could accurately reflect the final protein product after the translation and modification of transcriptional information, it failed to capture the upstream or intermediate phases of gene expression.

In addition to the utilisation of SnRNA-seq analyses, ferroptosis which was recognised as a newly discovered form of regulated cell death was comprehensively analysed and studied in our study [46]. However, to date, there is still no literature on the mechanisms of associated ferroptosis in high-altitude hypoxic TBI [47]. The existing literature focused only on the pathogenesis of ferroptosis after TBI in the plain area [48]. A previous study by our group showed that ferroptosis was induced after TBI and the inhibition of ferroptosis would protect against TBI in a CCI mouse model [1]. A recent study explored that nuclear factor erythroid-derived 2-related factor 2 (NRF2), the predominant transcription factor regulating oxidative stress and neuroinflammation in TBI, is neuroprotective against TBI-induced ferroptosis [49]. Nevertheless, these literatures have only employed some immunolabelling methods to validate the mechanism of ferroptosis in TBI [50]. The advantage of our study was that we firstly analyzed the involvement of ferroptosis in the pathogenesis of TBI using a snRNA-seq approach, and later validated the snRNA-seq results with immunolabelling methods. Comparing the TBI-induced astrocytic ferroptosis in plain area from Cheng’s study with TBI-induced astrocytic ferroptosis in high-altitude from our study showed that iron deposition was not detected in plain area due to a strong ability for iron transportation, but the apparently observable ferroptosis in high-altitude was suspected to be related to hypoxia [51, 52]. The characteristics of the iron metabolism in astrocytes after TBI still require further investigation.

Our findings also showed a dramatic decrease in neuronal function in high-altitude TBI, but it was currently unclear how increased ferroptosis in astrocytes impacts neuronal function in high-altitude TBI. Kain Seo confirmed that the astrocytic Ca²^+^ elevation promoted social avoidance and anxiety, as well as increased heart rate in socially stressed male mice by inhibiting neighboring neurons [53]. Inhibiting astrocyte ferroptosis can suppress release of ferroptosis-related inflammatory factors, as well as prevent neuronal degeneration in rats with spinal cord injury [54]. In the existing literature of plain TBI, astrocytes lose the ability to regulate the iron level of neurons by inhibiting the TfR1/DMT1 pathway after ferroptosis, resulting in impaired neuronal function [55]. In our study, the ROS of the high-altitude TBI were significantly elevated, which might impair neuronal function, but the underlying molecular mechanism still needed to be further exploration.

Through comprehensive analysis of snRNA-seq and immunolabelling approach, we found that Bach1 may be a hub site for postinjury regulation of high-altitude hypoxia [53, 54]. Generally speaking, Bach1 has a key regulatory role in the production of reactive oxygen species, cell cycle, heme homeostasis, hematopoiesis, and immunity and has been shown to suppress ischemic angiogenesis and promote breast cancer metastasis [55, 56]. However, so far, neither TBI in plains area nor high-altitude hypoxic TBI has reported about Bach1 [57]. In the present study, we verified the function and effect of Bach1 in high-altitude hypoxic TBI for the first time, and found that Bach1 knockdown could effectively reversed astrocytic ferroptosis induced by high-altitude hypoxic TBI. A previous study showed that Bach1-deficient mice was more resistant to the oxidative stresses associated with hyperoxia lung injury, nonalcoholic steatohepatitis, and cardiovascular disease, as well as bleomycin-induced pulmonary fibrosis [58, 59]. A recent study confirmed that Bach1 overexpression enhanced the production of reactive oxygen species (ROS) from the mitochondria of endothelial cells and in the ischemic limbs of mice, which leaded to increase in apoptosis and decline in angiogenesis [60]. Although the results of these literatures are in agreement with ours, it can be seen out that there are still few reports of Bach1 regulating ferroptosis.

There are several limitations in our study. First, we focused exclusively on the cortical damaged area, while changes in hippocampus may also account for the ferroptosis seen after high-altitude hypoxic TBI. In our study, although HE staining showed pathological specimens of the hippocampus, the hippocampal region was not studied thoroughly enough and lacked snRNA-seq results. Future studies on changes in snRNA-seq in hippocampus may help reveal the unique molecular mechanisms of pathogenesis after high-altitude hypoxic TBI that relate to the observed biological phenotypes. Second, based on snRNA-seq analysis, we identified that upregulation of Bach1 expression in astrocytes is a key factor in high-altitude hypoxia aggravating TBI and validated this through relevant experiments. But in fact, there are other types of cells and gene expression that have undergone significant changes, which may also play a key role in high-altitude hypoxia aggravating TBI. We will continue to conduct in-depth research in future work. Finally, in terms of mechanism, we have only preliminarily verified the role of Bach1 in the high-altitude hypoxic TBI, and its specific regulatory mechanism needs further in-depth verification and research. In the future, we will continue to conduct comprehensive studies in a model of high-altitude hypoxic TBI and strive to fully reveal the pathological mechanism.

In conclusion, our study found that high-altitude hypoxia upregulates the expression of Bach1 in astrocytes, which in turn induces ferroptosis in astrocytes to exacerbate brain injury in a mouse TBI model. Knockdown of Bach1 could alleviate high-altitude hypoxia aggravating TBI in mice. This study lays a theoretical foundation for a deeper understanding of the mechanisms of high-altitude traumatic brain injury and targeted intervention treatment based on this foundation.

## Materials and Methods

### Animals

The 6–8 w male mice used in this experiment were all C57BL/6 strains, were purchased from the Animal Experiment Center of the Air Force Medical University. The high-altitude low oxygen environment was simulated using a small low-pressure oxygen chamber capable of mimicking altitudes up to 10,000 m, as shown in Fig. 1A. The chamber’s main working principle involved vacuum pumping to create a low-pressure, low-oxygen environment, with an internal pressure sensor used to simulate different altitudes. This experiment simulated altitudes of 4000 m, 6000 m, and 8000 m respectively. The chamber’s temperature was maintained at around 25 °C, with a humidity level of 60–70%. During the feeding period, the mice had free access to water, and ascent and descent speeds were set 5 m/s. 6 mice in each group were randomly allocated for behavioral testing.

### Model of traumatic brain injury (TBI)

The TBI model was created using a controlled cortical impact model (CCI), as shown in Supplemental Fig. 1A. In this model, mice were anesthetized with inhalation of 3% isoflurane (RWD Life Science, Shenzhen, China). Anesthesia was maintained with 2% isoflurane. After removing the hair on the mouse head with a hair clipper, it was thoroughly disinfected by three times. The skin was then incised using tweezers and tissue scissors, and the skull periosteum and attached muscles were peeled back to expose the underlying area. Subsequently, a desktop dental grinder was used to remove a skull window of approximately 3.5 mm, with the center of the window positioned 2 mm to the right of the sagittal suture and 1 mm window positioned the anterior fontanelle (Supplemental Fig. 1B). The mice in the high-altitude TBI group were fed at simulated altitudes of 4000 m, 6000 m, and 8000 m for 3 days. TBI models were created using an electromagnetically driven impactor (Hatteras Instruments Inc. PinPointTM PCI3000, Grantsboro, NC, USA). After the models were completed, the mice continued to be housed at the simulated altitudes of 4000 m, 6000 m, and 8000 m for 7 days. The control group only underwent craniotomy without impact. The CCI striking instrument parameters were set as follows: impact velocity of 3.0 m/s, impact depth of 1 mm, and impact duration of 180 ms. The striking probe was adjusted so that its farthest end gently adhered to the dura mater beneath the skull bone window, and then pressed the start button to strike. Immediately after the impact, the mice were placed under a respirator mask to inhale oxygen, the wound was disinfected again, and then sutured. Additionally, the mice were placed on a thermal blanket to maintain normal body temperature.

### Neurobehavioral training and evaluation

Neurological deficits were assessed using well-established tests, including the modified neurological severity score (mNSS), rotarod test and elevated plus maze test. Assessments were conducted at baseline and daily after TBI or sham surgery by two investigators who were blinded to the experimental design.

### Modified neurological severity scores

This study used the Modified Neurological Severity Scores (mNSS), which comprehensively evaluates various indicators of motor, sensory, reflex, and balance functions in injured mice. The scores are negatively correlated with neurological function, with higher scores indicating more severe brain trauma. A score of 0 indicates a fully functional mouse. The mNSS scoring scale is shown in Table 3.

**Table 3:** Modified neurological severity score (mNSS).

Tests	Points
Motion tests	
Raising rat by tail (normal=0; maximum=3)	
Flexion of forelimb	1
Flexion of hindlimb	1
Head moved>10 degree vertical axis within 30 s	1
Walking rat on floor (normal=0; maximum=3)	
Normal walk	0
Inability to walk straight	1
Circling toward the paretic side	2
Falls down to paretic side	3
Sensory tests (normal=0; maximum=2)	
Placing test (visual and tactile test, try 5 times and have not moved more than 3 times)	1
Proprioceptive test (deep sensation, pushing the paw against the table edge to stimulate	
limb muscles, try 5 times and have not moved more than 3 times)	1
Beam balance test (normal=0; maximum=6)	
Balances with steady posture (>60 s)	0
Grasps side of the beam	1
Hugs beam and 1 limb falls down from beam	2
Hugs beam and 2 limbs falls down from beam, or spins on beam (>60 s)	3
Attempts to balance on beam but falls off (>40 s)	4
Attempts to balance on beam but falls off (>20 s)	5
Falls off, no attempt to balance or hang on the beam (<20 s)	6
Lack of reflexes and abnormal movement (normal=0; maximum=4)	
Auricle reflex (head shaking when touching the auricle)	1
Corneal reflex (blinking action when a mouse’s cornea is lightly touched)	1
Panic reflex (noise does not induce movement)	1
Epilepsy, myoclonus, dystonia	1
Maximum Points	18

### Rotarod test

Motor function was detected by the rotarod test equipment (IITC Life Science, Woodland Hills, CA, USA). At the beginning of the experiment, the starting speed was set to 4 revolutions per minute, which was gradually increased to 40 revolutions per minute over 5 min. The automatic timer was activated when the mouse began crawling and rotating on the turnbar. The timer stopped automatically when the mouse fell off the turnbar, detected by the infrared device. Before modeling, all mice were intermittently trained on the rotating pole for 3 days, and the enrolled mice were required to remain on the turnbar for at least 1 minute. The experimental mice underwent behavioral testing immediately after leaving the cabin. Using the discontinuous multiple measurement method, each test was repeated three times, and the average value was calculated.

### Elevated plus maze test

The elevated plus maze was placed in a soundproof room with low light illumination. Before starting the test, the cross platform was cleaned with 75% alcohol. The mouse was then placed with its back to the experimenter, facing the open arm. The video tracking system was activated on the central platform of the elevated maze. The system automatically recorded the residence time of mice in both open and closed arms, as well as the distances they entered in each arm. The testing time for each mouse was 5 minutes. The operator avoided unnecessary movement and noise throughout the testing process, ensuring that each mouse was placed in a consistent position on the platform. After the experiment, system software (Smart 3.0, Panlab, MA, USA) was used to analyze indicators such as the time spent and distance traveled in the open arm for each group of mice.

### Tissue processing

After completing the behavioral experiments, the mice were anesthetized with 3% isoflurane (RWD Life Science) and maintained under anesthesia with 2% isoflurane. The mice were then sacrificed by perfusion with ice-cold 0.01 M phosphate-buffered saline (PBS) (pH 7.4), followed by fixation with 4% paraformaldehyde. Gross brain specimens were collected. Fresh brain tissue was used for Western blot (WB) analysis and single-cell sequencing. Next, the brains were removed and postfixed for 4 h, followed by dehydration in 30% sucrose solution for 48 h.

### Single-nuclear RNA sequencing (snRNA-seq) analysis

All mice underwent snRNA-seq analysis using brain tissue from the right hemisphere, collected 7 days after injury or control injury. SnRNA-seq and subsequent analyses were commissioned by New Geyuan Biotechnology Co., LTD (Nanjing, Jiangsu, China).

Single-cell suspensions (2 × 10^5^ cells/mL) in PBS (HyClone) were loaded onto a microwell chip using the Singleron Matrix^®^ Single Cell Processing System. Barcoding Beads were subsequently collected from the microwell chip, followed by reverse transcription of the mRNA captured by the Barcoding Beads to obtain cDNA, which was then amplified by PCR. The amplified cDNA was then fragmented and ligated with sequencing adapters. The snRNA-seq libraries were constructed using the GEXSCOPE^®^ Single Cell RNA Library Kits (Singleron). Libraries were diluted to 4 nM, pooled, and sequenced on an Illumina novaseq 6000 platform with 150 bp paired end reads. Raw reads were processed into gene expression matrixes using CeleScope (https://github.com/singleron-RD/CeleScope) v1.9.0 pipeline. Quality control, dimensionality reduction, and clustering were performed using Scanpy (v1.8.2) under Python 3.7.

To identify differentially expressed genes (DEGs), we used the Seurat FindMarkers function based on Wilcox likelihood-ratio test with default parameters, and selected the genes expressed in more than 10% of the cells in a cluster and with an average log (Fold Change) value greater than 0.25 as DEGs. For the cell type annotation of each cluster, we combined the expression of canonical markers found in the DEGs with knowledge from literatures, and displayed the expression of markers of each cell type with heatmaps/dot plots/violin plots that were generated with Seurat DoHeatmap, DotPlot, Vlnplot function. Doublet cells were identified as expressing markers for different cell types, and removed manually. The cell type identity of each cluster was determined with the expression of canonical markers found in the DEGs using SynEcoSys database. Heatmaps/dot plots/violin plots displaying the expression of markers used to identify each cell type were generated by Seurat v3.1.2 DoHeatmap/DotPlot/Vlnplot. To investigate the potential functions of DEGs, the Gene Ontology (GO) and Kyoto Encyclopedia of Genes and Genomes (KEGG) analysis were used with the clusterProfiler R package (v3.16.1). Transcription factor network was constructed by pyscenic (v0.11.0) using snRNA expression matrix and transcription factors in AnimalTFDB.

### Western blotting

The fresh brain tissue was collected and homogenized in ice-cold RIPA buffer containing protease inhibitors (Glpbio, Montclair, CA, USA, Cat# GK10014) and phosphatase inhibitors (Glpbio, Cat# 23227 GK10012) to isolate total protein. The protein samples were quantified using a BCA assay kit (Cat# 23227, Thermo Fisher Scientific, Waltham, MA, USA). Next, the samples were loaded onto an 8% SDS-PAGE gel (Cat# M00661, Genscript, Nanjing, China) separated by electrophoresis. Subsequently, the proteins were transferred to PVDF membranes (Millipore, Darmstadt, Germany) that were blocked with 5% nonfat milk for 1 hour and incubated with the following primary antibodies overnight at 4 °C: anti-Bach1 (rabbit, 1:1000, 14018-1-AP, Proteintech, Wuhan, China); anti-Fth1 (rabbit, 1:1000, 10727-1-AP, Proteintech, Wuhan, China); beta-Tubulin (rabbit, 1:2000, 10068-1-AP, Proteintech, Wuhan, China); Then, the membranes were incubated with a horseradish peroxidase-conjugated secondary antibody (goat anti-rabbit IgG, 1:5000, GB23303, Servicebio, Wuhan, China) for 1 hour. Finally, the membranes were incubated with a chemiluminescence reagent (RM00021, ABclonal) and imaged with a CHEMIL-MAGER chemiluminescence imaging system (Bio-Rad, Hercules, CA, USA). ImageJ software (fiji-2.11.0, NIH, Bethesda, MD, USA) was used to measure the intensity of each band. Relative protein expression was normalized to GAPDH.

### Hematoxylin-Eosin (HE) staining and immunofluorescence staining

Mice were anesthetized with 3% isoflurane (RWD Life Science), followed by cardiac perfusion with physiological saline and 10% paraformaldehyde to fix brain tissue. The fixed tissue was made into paraffin sections (Leica CM 1950, Wetzlar, Germany). The specific steps of HE staining were as follows: tissue slices were dewaxed with xylene, treated with gradient alcohol, washed with tap water, stained with hematoxylin dropwise for 5 min, phenolized with hydrochloric acid ethanol for 5 s, returned to blue with running water for 90 s, and stained with eosin for 2 min. Gradient ethanol dehydration, xylene transparency, and neutral gum sealing were observed and photographed under a microscope. The specific steps of immunofluorescence staining were as follows: the tissue sections were collected, placed on slides, washed in 0.01 M PBS (three times for 10 min each), and blocked with 5% bovine serum albumin solution and 0.3% Triton-X100/PBS for 1 hour. Then tissue sections were incubated overnight at 4 °C with primary antibody: Bach1 (mouse, 1:1000, Cat# sc-271211, Servicebio, Wuhan, China); Fth1 (rabbit, 1:3000, Cat# ab75973, Servicebio, Wuhan, China), followed by incubation at room temperature for 3 h with secondary antibody (donkey anti-mouse, Alexa FluorTM 594, 1:8000, Cat# ab150108, Servicebio, Wuhan, China). The primary and secondary antibodies were diluted in 5% bovine serum albumin and 0.1% Triton-X100/PBS. Cellular nuclei were stained by incubating the slides for 10 min in 4’,6-diamidino-2-phenylindole (DAPI) (1:1000, Sigma, St. Louis, MO, USA, D9564) diluted in PBS. Finally, the slides were coverslipped with 50% glycerin mounting medium.

### ELISA (enzyme linked immunosorbent assay)

The HIF-1α concentration was measured according to previous experiments. Five milligrams of tissue were transferred into a silicified glass tube, cut into 1 mm^3^ pieces and incubated in 0.4 mL of PBS at 4 °C. After 20 min, the supernatant was separated from the tissue fragments, which were then homogenized in 0.4 mL of a (1:1) methanol/water mixture. The amount of HIF-1α in the supernatant was measured by a mouse HIF-1α ELISA kit (MEIKE Jiangsu Sumeike Biological Technology Co., Ltd) according to the instructions. The amount of HIF-1α was normalized to the protein content.

### Evans blue (EB) extravasation test

To measure blood-brain barrier (BBB) permeability, 2% Evans Blue (4 mL/kg) in sterile saline was injected through the tail vein 1 h before the animals were sacrificed. The mice were transcardially perfused with saline, and their brains were dissected and weighed. The samples were then homogenized in PBS (1 mL/300 g), sonicated for 2 min, and centrifuged at 15,000 rpm for 5 min at 4 °C, and the supernatant was then collected in aliquots. Next, 500 μL of 50% trichloroacetic acid was added to each 500 μL of supernatant and incubated overnight at 4 °C. Finally, these samples were centrifuged at 15,000 rpm for 30 min at 4 °C. The samples were detected with a spectrometer at 610 nm and quantified using a standard curve that was normalized to tissue weight (μg/g). To assess the fluorescence intensity, the brains were removed in preparation for coronal brain sectioning. Red autofluorescence of EB was observed on the slides as previously described. The mean red autofluorescence of EB was evaluated by 2 observers blinded to the mouse treatment groups.

### Brain water content

After anaesthetization and euthanasia, the mice were decapitated one week after TBI, and their brains were immediately removed. Each brain was weighed immediately to determine the wet weight and then dried for 24 h at 100 °C to obtain the dry weight. The percentage of brain water content was calculated as follows: (wet weight-dry weight)/wet weight × 100%. The percentage of water content was calculated by 2 trained investigators who were blinded to the animal grouping.

### ROS Detection

Tissue ROS was determined by a tissue ROS detection kit (Baiolaibo Technology Co., LTD., Beijing, China). According to the kit instructions, 50 mg of fresh right cortical tissue was accurately weighed, washed with PBS buffer, centrifuged at 4 °C for 3 min, and the precipitation was discarded and the supernatant was taken. 200 μL homogenate supernatant and 2 μL of dihydroethidium (DHE; Sigma-Aldrich) probe were added into the black 96-well plate respectively. After incubation at 37 °C for 15 ~ 30 min, the fluorescence intensity was measured at the excitation wavelength of 488-535 nm and the emission wavelength of 610 nm. In contrast, the protein concentration of the homogenate supernatant was measured with a BCA Protein Assay Kit (Thermo Scientific, MA) according to the manufacturer’s instructions. Tissue ROS intensity was represented as the fluorescence intensity normalized to milligrams of protein.

### Infection with recombinant adeno-associated virus

The recombinant rAAV2/5-GFAP-EGFP-5′miR-30a-shRNA Bach1-3′miR-30a-WPREs and rAAV2/5-GFAP-EGFP-5′miR-30a-shRNA ctrl-3′miR-30a-WPREs control viruses were generated by BrainVTA Co., Ltd (Wuhan, China). For in vivo infection, an AAV downregulated Bach1 (200 nL, 1.2 × 10^12^ viral genomes/mL) or a control virus (200 nL, 1.3 × 10^12^ viral genomes/mL) was administered via intracortical injection using a slow infusion rate of 100 nL/min. The injection sites were 4 points (anteroposterior (AP): −0.4 mm, mediolateral (ML): 0.5 mm, dorsoventral (DV): −1.27 mm; AP: −1.6, ML: 0.6/1.5 mm, DV: −1.00 mm, AP: −0.4 mm, ML: 1.5 mm, DV: −1.53 mm). TBI was induced 4 weeks after recovery from the intracortical virus injection.

### Statistics

All experimental data were obtained from three or more independent repeated experiments, processed using the GraphPad Prism 9.0 software package, and quantitative data were expressed as mean ± standard deviation (SD). The Shapiro-Wilk normality test was used to test the normality of the variable distribution. The comparison between the two groups was conducted using independent sample t-tests. For multiple group comparisons, one-way ANOVA followed by Tukey’s multiple comparisons test or two-way ANOVA followed by Tukey’s multiple comparisons test were used for statistical comparisons. The comparison of survival curves was conducted using the Log-rank test. Single**-**Cell distribution comparisons between two groups were performed using unpaired two-tailed Wilcoxon rank-sum tests. *P* < 0.05 indicated significant differences, and ns indicates not significant.

## Supplementary information

pyscenic-Astrocytes

Astrocytes-LA_TBIvsHA_TBI.diffexpressed

Supplemental Figure Legends

Supplemental Figure 1

Supplemental Figure 2

Supplemental Figure 3

original western blots

## Data Availability

Data is provided within the manuscript or supplementary information files.
