# Supplementary material for: High-altitude hypoxia aggravated neurological deficits in mice induced by traumatic brain injury via BACH1 mediating astrocytic ferroptosis
Source: Cell Death Discov. 2025 Feb 5;11:46. doi: 10.1038/s41420-025-02337-8 (PMC11794473; doi:10.1038/s41420-025-02337-8)
Supplement: Supplementary file 3 — Supplemental Figure Legends [file 41420_2025_2337_MOESM3_ESM.docx]

**Supplemental** **Figure Legends:**

**Supplemental** **Figure 1. The behavioral results of high-altitude TBI.** (A) The CCI device for TBI in mice. (B) The size of the cranial bone removed in mice. (C-E) The mNSS of varying altitude TBI group comparing with respective sham group. (C) Comparison of mNSS between the HAS-4km and HAT-4km group (unpaired t-test, t = 19.20, *P* < 0.0001), (D) Comparison of mNSS between the HAS-6km and HAT-6km group (unpaired t-test, t = 28.27, *P* < 0.0001), (E) Comparison of mNSS between the HAS-6km and HAT-6km group (unpaired t-test, t = 24.98, *P* < 0.0001). (F-H) The rotarod latency of varying altitude TBI group comparing with respective sham group. (F) Comparison of rotarod latency between the HAS-4km and HAT-4km group (unpaired t-test, t = 55.71, *P* < 0.0001), (D) Comparison of mNSS between the HAS-6km and HAT-6km group (unpaired t-test, t = 63.69, *P* < 0.0001), (E) Comparison of mNSS between the HAS-6km and HAT-6km group (unpaired t-test, t = 58.39, *P* < 0.0001). (I) Survival rate curves of mice in HAT-4km, HAT-6km, and HAT-8km groups. N=6. (Log-rank test, χ^2^= 11.89, ^&^*P* =0.0026 versus the HAT-4km group. χ^2^= 4.572, ^$^*P* < 0.0325 versus the HAT-6km group).

**Supplemental** **Figure 2. The histopathological injury of high-altitude TBI.** (A) The brain gross specimens from the HAS-4km group, the HAS-6km group and the HAS-8km group. (B) The brain gross specimens from the HAS-4km group, the HAS-6km group and the HAS-8km group after EB extravasation test. (C) HE staining of the right side of hippocampus and cortex in different groups, scale=50 μm. (D-F) The brain water content of varying altitude TBI group comparing with respective sham group. (D) Comparison of brain water content between the HAS-4km and HAT-4km group (unpaired t-test, t = 10.83, *P* < 0.0001), (E) Comparison of brain water content between the HAS-6km and HAT-6km group (unpaired t-test, t = 15.10, *P* < 0.0001), (F) Comparison of brain water content between the HAS-8km and HAT-8km group (unpaired t-test, t = 17.04, *P* < 0.0001). (G-I) The EB content of varying altitude TBI group comparing with respective sham group. (G) Comparison of EB content between the HAS-4km and HAT-4km group (unpaired t-test, t = 17.34, *P* < 0.0001), (E) Comparison of EB content between the HAS-6km and HAT-6km group (unpaired t-test, t = 28.23, *P* < 0.0001), (F) Comparison of EB content between the HAS-8km and HAT-8km group (unpaired t-test, t = 40.02, *P* < 0.0001).

**Supplemental** **Figure 3. The outcomes of differential gene enrichment in inhibitory neurons.** (A) Heatmap plots showing representative differentially expressed genes between the LA-TBI and HA-TBI groups of inhibitory neurons. (B and C) The top 10 GO enrichments in BP, CC, MF in up-regulated (B) and down-regulated (C) DEGs in inhibitory-neurons. Each node signaled a GO term, and its size represented the gene number.
