# Supplementary figures and images for: High-altitude hypoxia aggravated neurological deficits in mice induced by traumatic brain injury via BACH1 mediating astrocytic ferroptosis
Source: Cell Death Discov. 2025 Feb 5;11:46. doi: 10.1038/s41420-025-02337-8 (PMC11794473; doi:10.1038/s41420-025-02337-8)

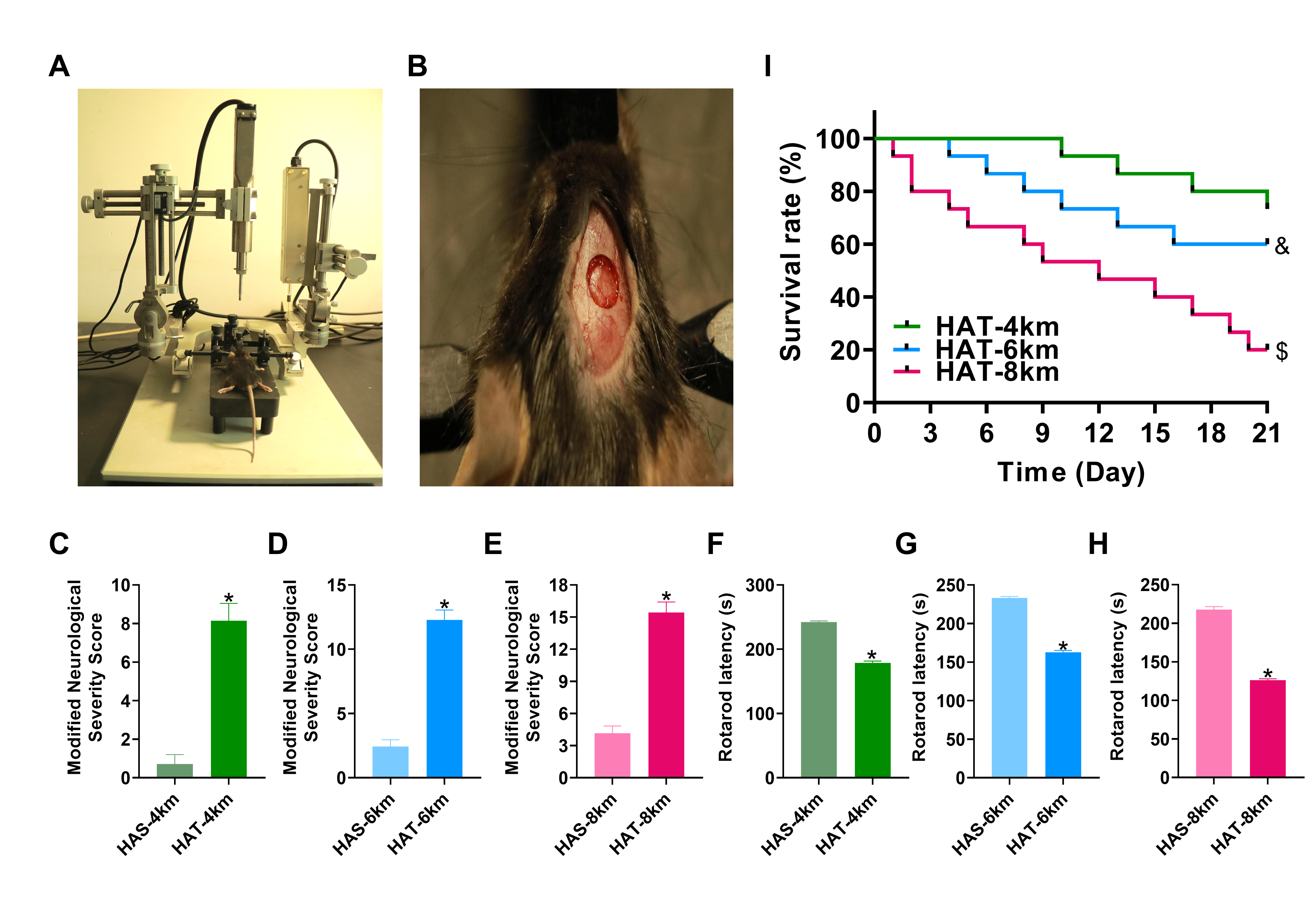

Supplement: Supplementary file 4 — Supplemental Figure 1 [file 41420_2025_2337_MOESM4_ESM.tif]

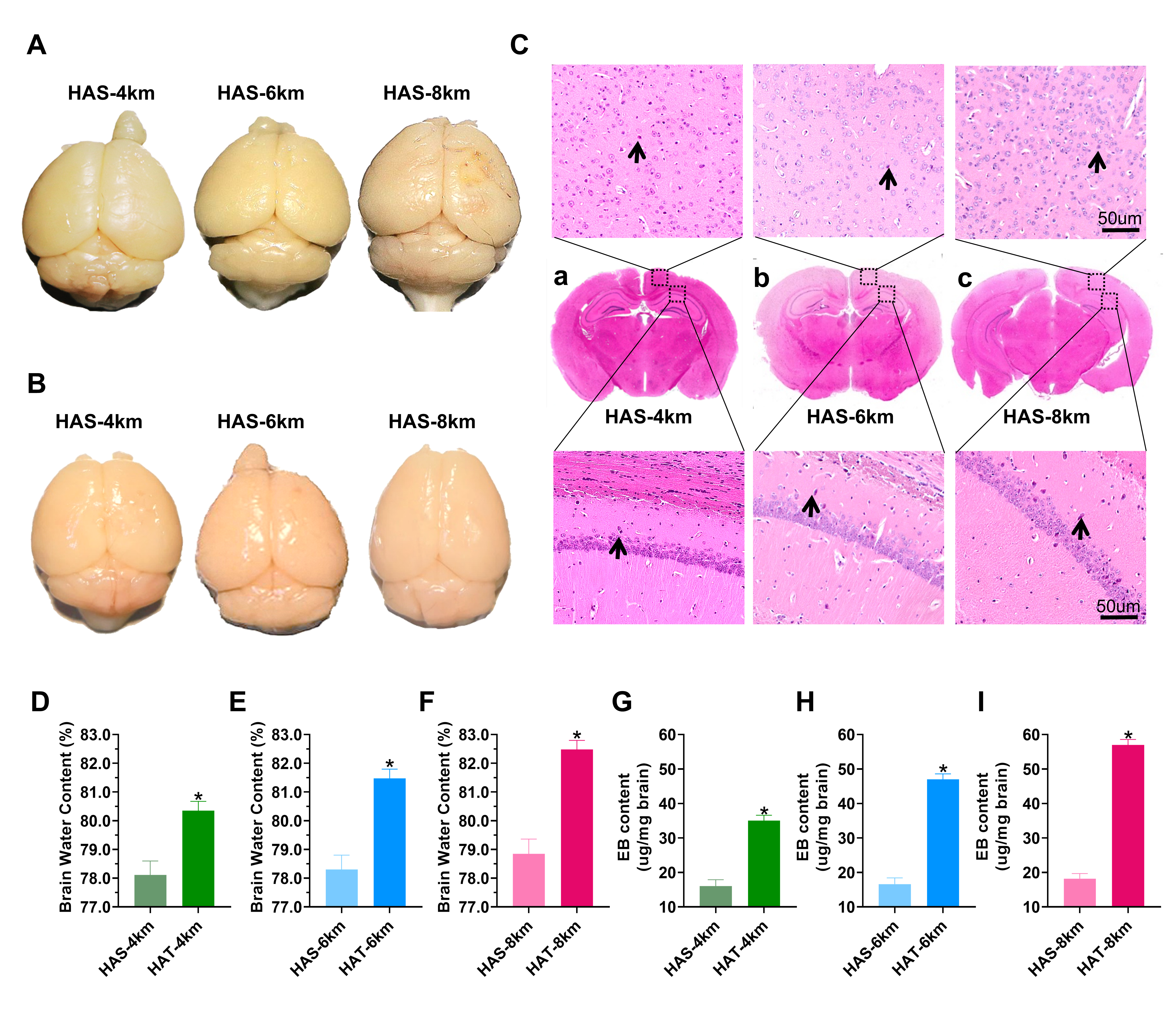

Supplement: Supplementary file 5 — Supplemental Figure 2 [file 41420_2025_2337_MOESM5_ESM.tif]

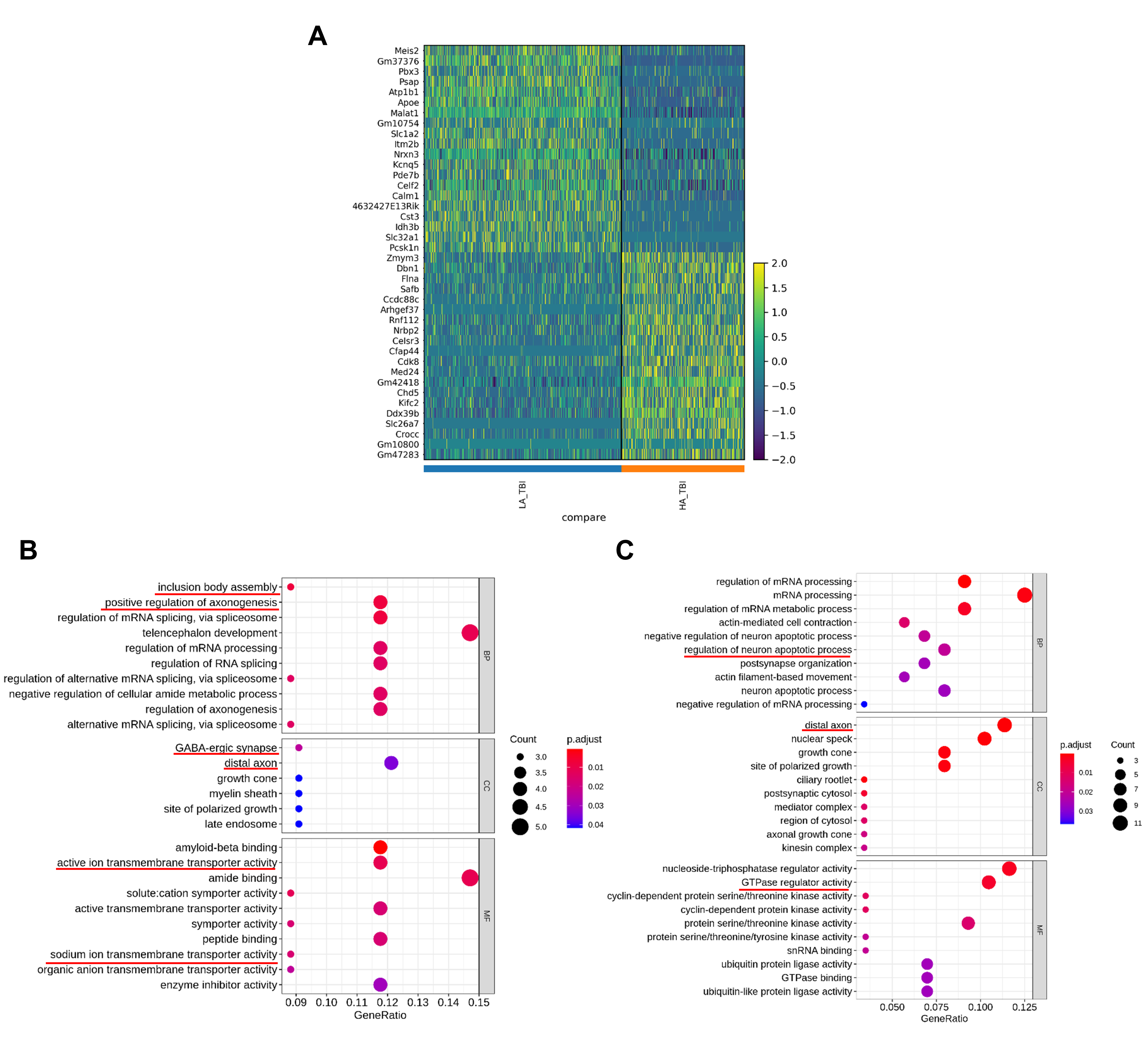

Supplement: Supplementary file 6 — Supplemental Figure 3 [file 41420_2025_2337_MOESM6_ESM.tif]

**Figure 5A**

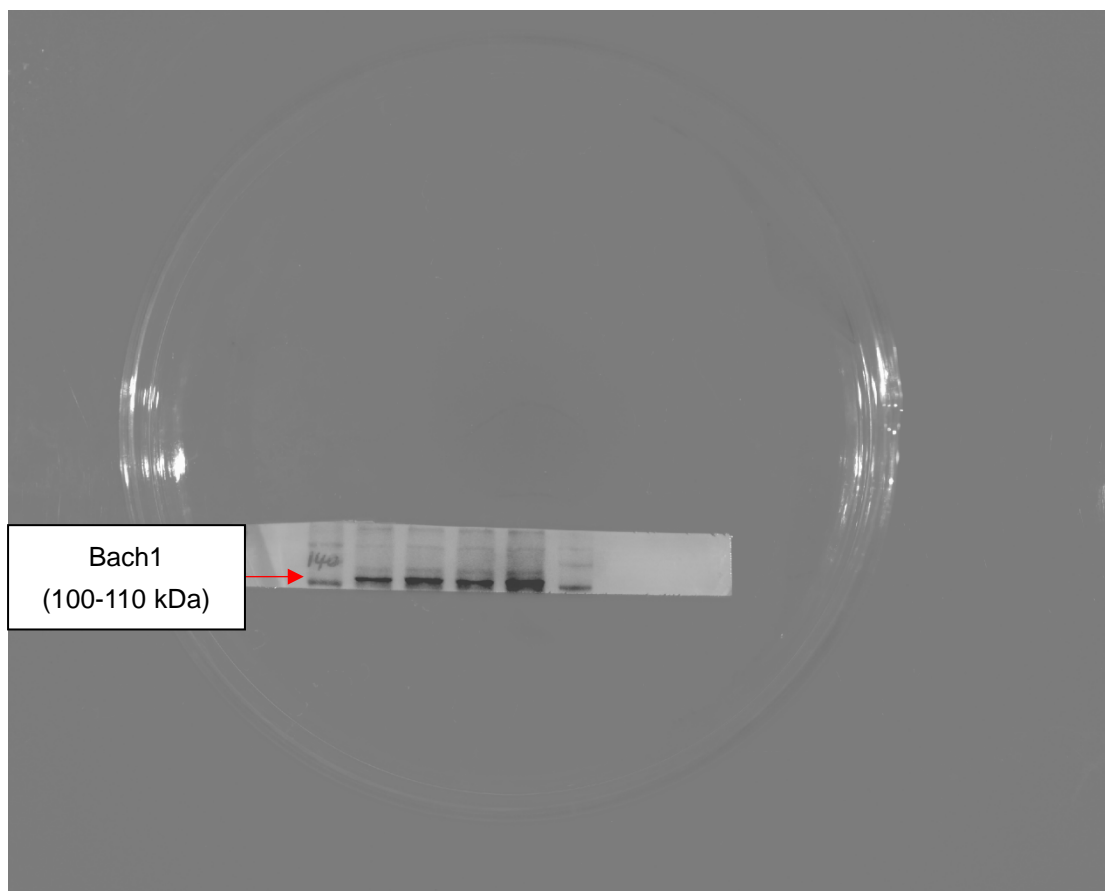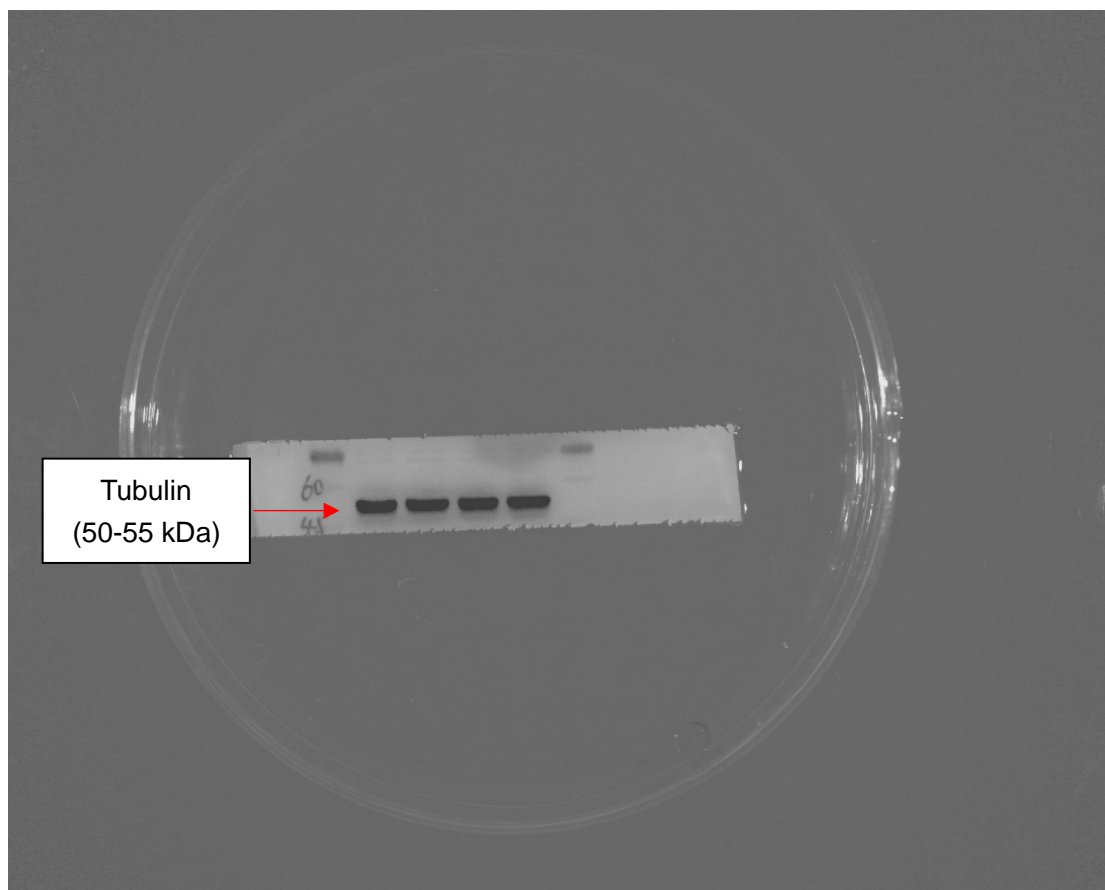

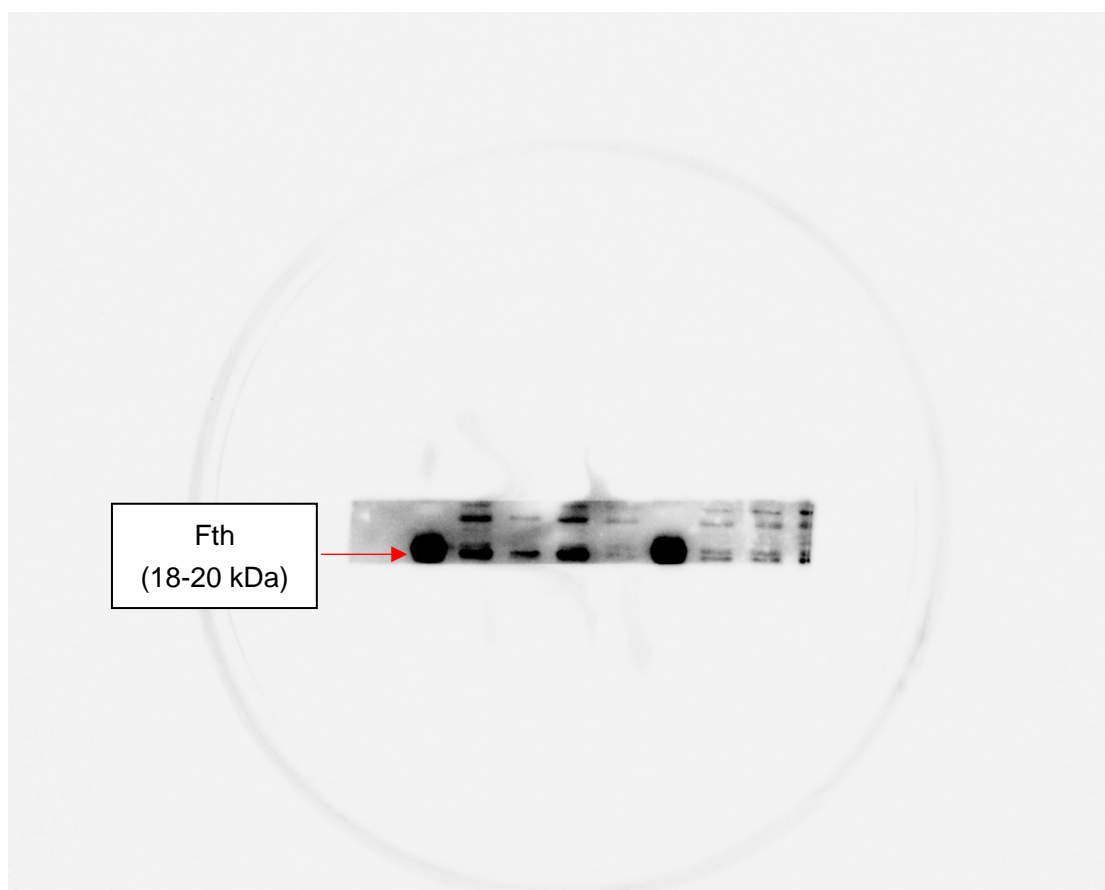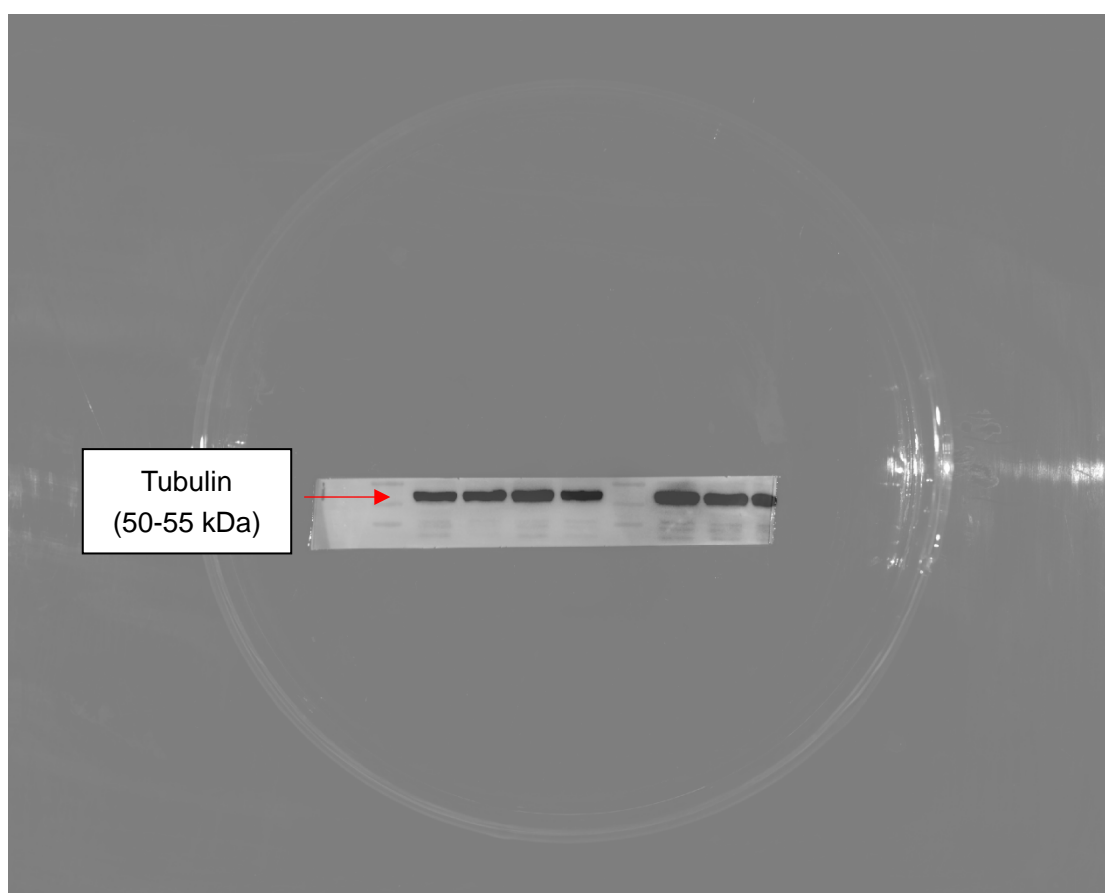

Fig 6B

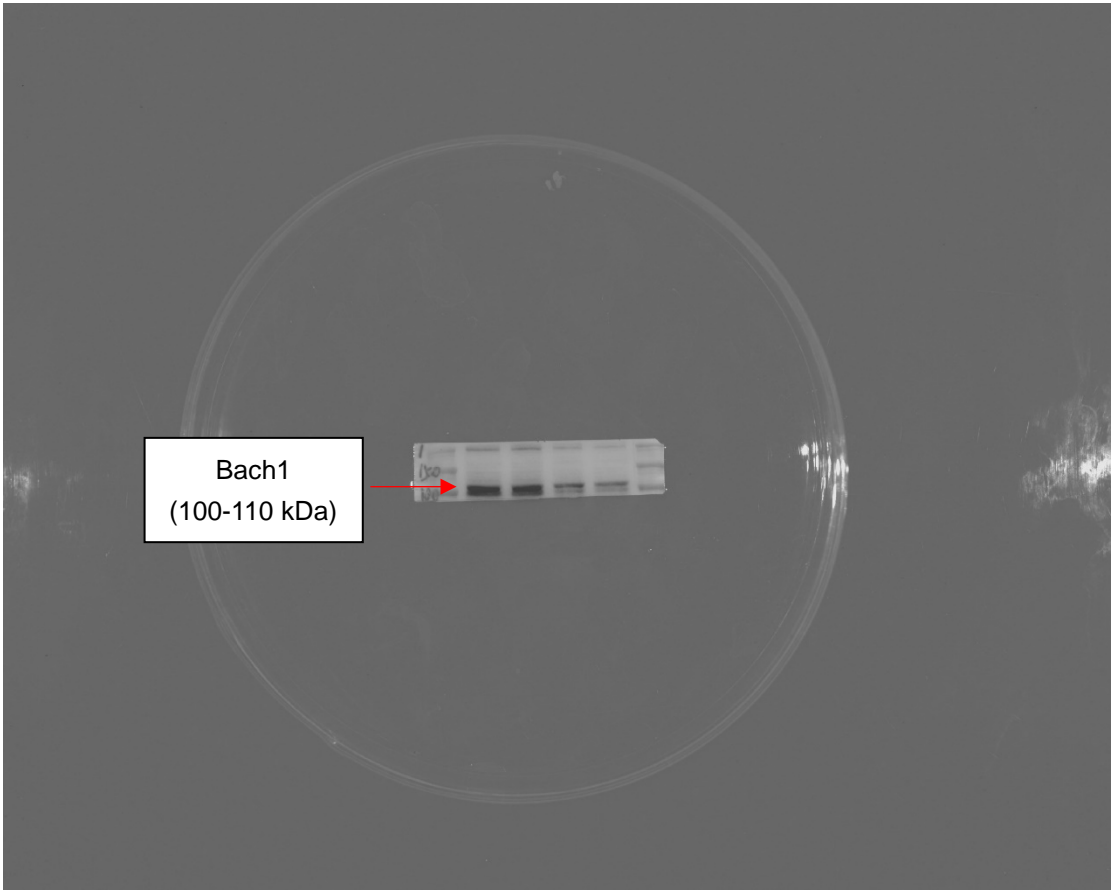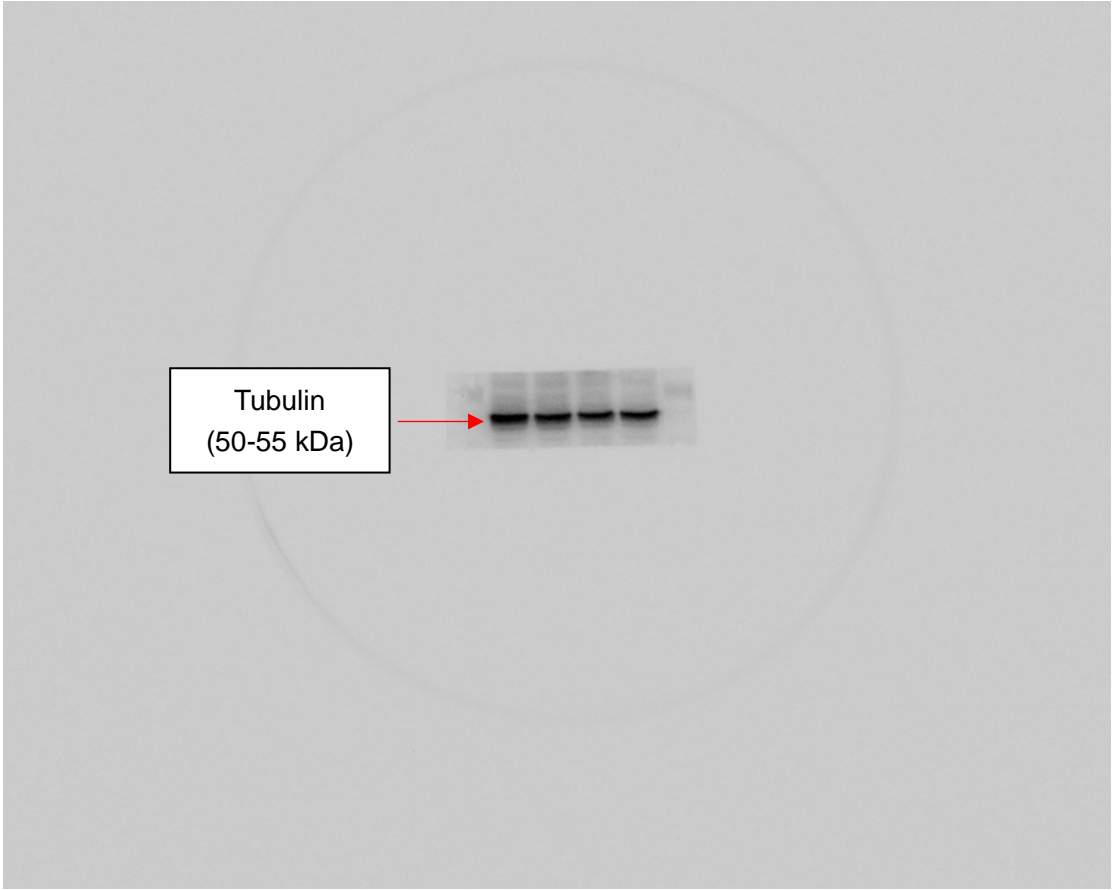

Fig 6J

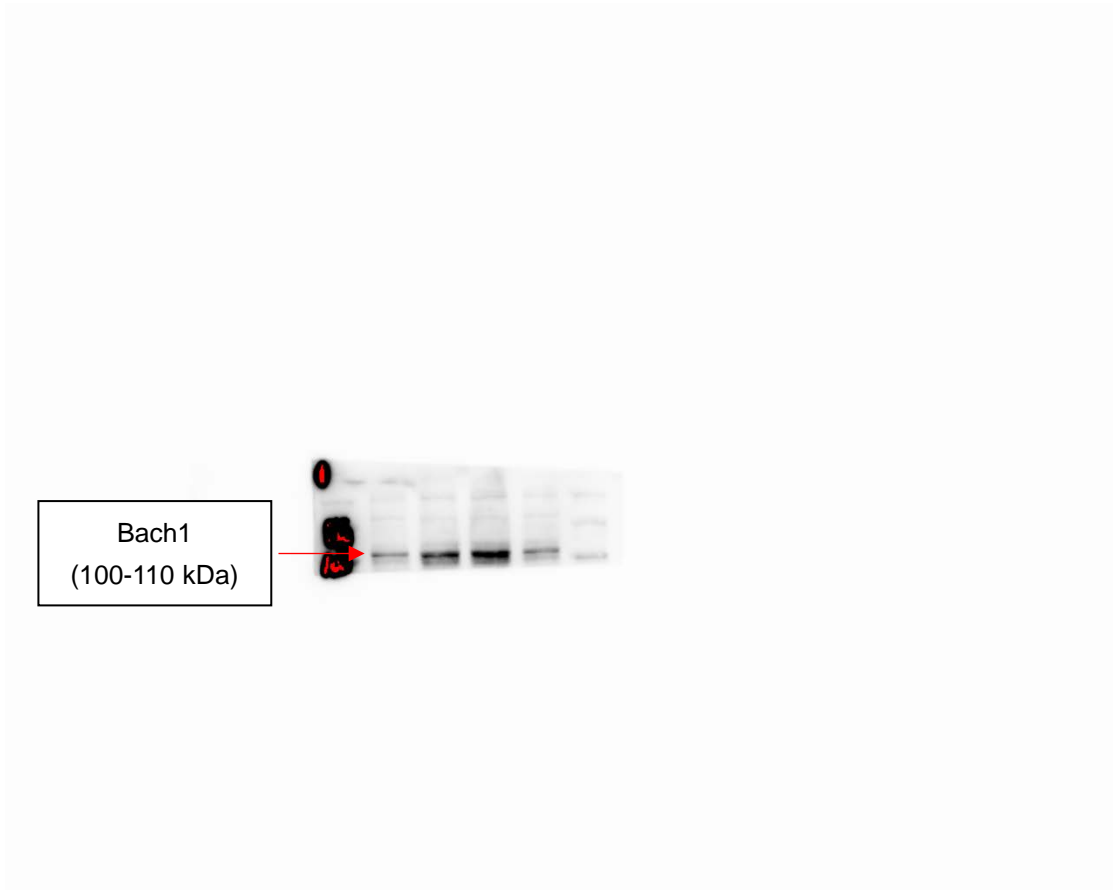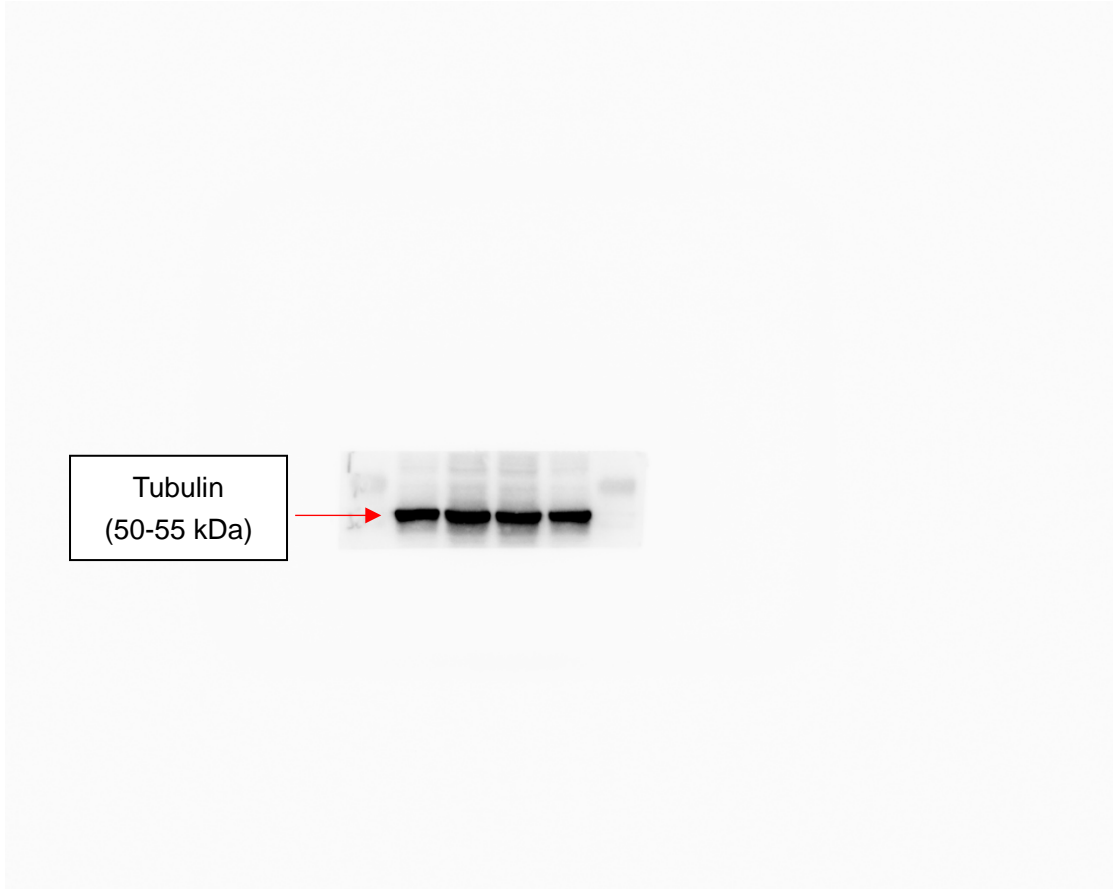

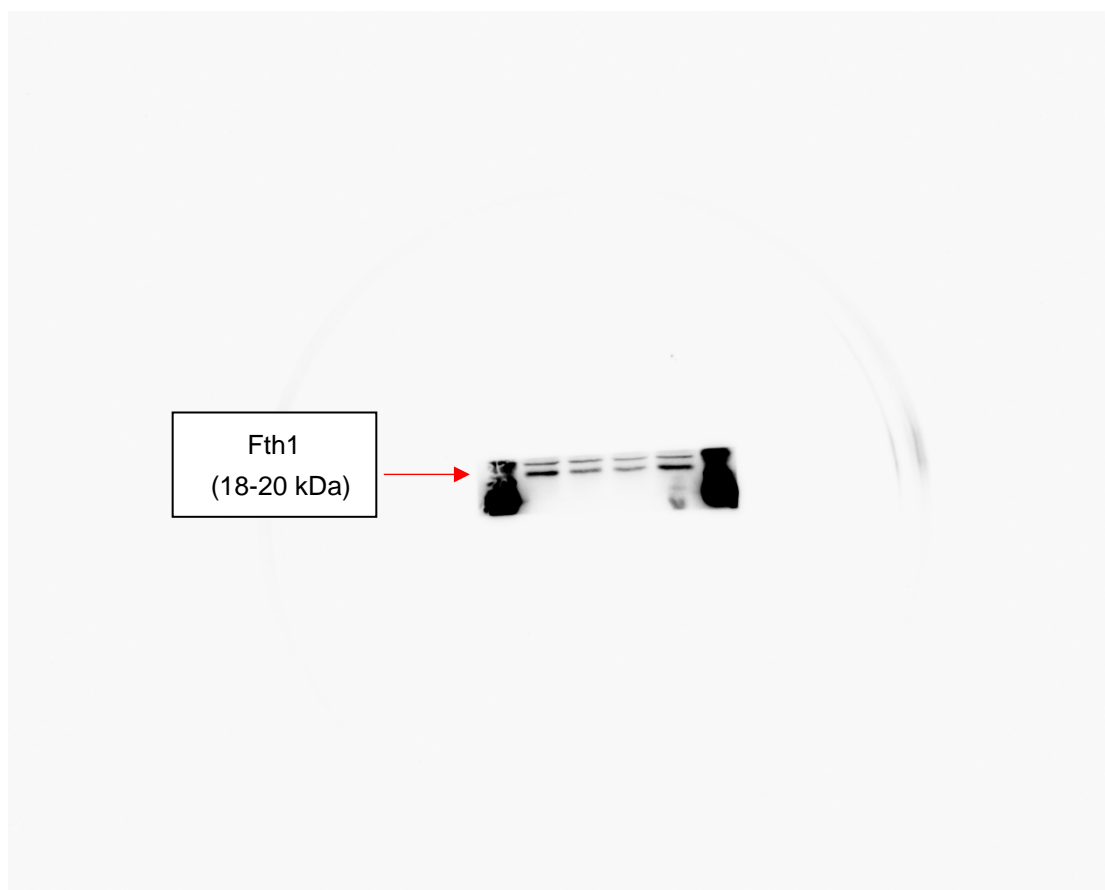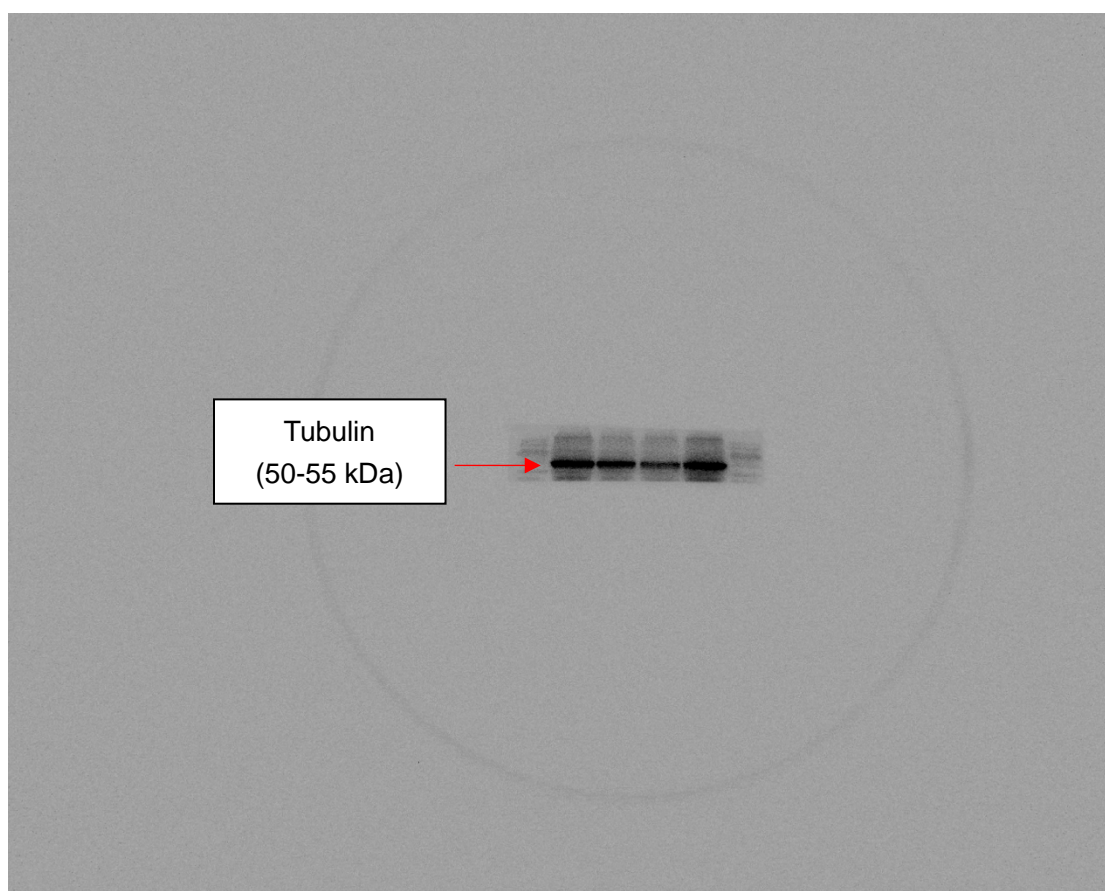

Supplement: Supplementary file 7 — original western blots [file 41420_2025_2337_MOESM7_ESM.pdf]
